# Alterations to fungal and viral pathogen infection in coinfected plant cultivars with diverse single-pathogen host resistances

**DOI:** 10.3389/fpls.2026.1809771

**Published:** 2026-09-09

**Authors:** Nuraizat Abidin, Martin J. Barbetti, Ming Pei You, Roger A. C. Jones

**Affiliations:** 1 School of Agriculture and Environment, The University of Western Australia, Crawley, WA, Australia; 2 Institute of Agriculture, The University of Western Australia, Crawley, WA, Australia

**Keywords:** *Brassica napus*, fungal-viral coinfection, host resistance, inter- and intra-pathogen interactions, leaf and plant age, *Leptosphaeria maculans*, resistance breaking strains, turnip mosaic virus

## Abstract

To examine how pathogen coinfection influences the efficacy of single-pathogen host resistances, we investigated the interactions between the fungus (*Leptosphaeria maculans*) and turnip mosaic virus in canola (*Brassica napus*). A resistance-breaking (RB) virus strain was inoculated onto the cotyledons of three cultivars. These cultivars featured distinct host resistance profiles: fungal single dominant gene resistance (SDGR) and polygenic resistance (PGR) along with viral temperature sensitive systemic invasion resistance (TSSIR) as well as a cultivar without fungus resistance serving as a control. Subsequently, systemically virus-infected true leaves of varying maturity were inoculated with RB or non-resistance-breaking (non-RB, i.e., wild-type) fungal strains both individually and in combination. This allowed assessment of the impacts of already established virus infection upon fungal disease severity and the reciprocal effects of subsequent fungus infection upon virus concentration (VC). In the youngest leaves, high VC values corresponded with the complete absence of fungal disease, whereas the lowest VC values in the oldest leaves were associated with maximum fungal disease severity. When the RB fungal strain was present alone, it overcame SDGR more effectively when the virus was absent. Conversely, presence of the RB fungal strain in SDGR-carrying plants decreased the VC values, regardless of the presence or absence of the non-RB fungus strain. In plants possessing both fungal PGR and viral TSSIR, regardless of fungal strain, fungal infection only occurred in the lower leaves of older plants which developed consistently low VC values like those in plants inoculated with the virus alone. In the fully fungus-susceptible cultivar, fungal disease levels were consistently lower in virus-infected than virus-mock-inoculated plants. However, in coinfected plants of this cultivar, VC values increased or decreased depending upon whether the fungal RB or non-RB strain was inoculated first. By examining virus–fungus pathogen interactions across a range of host resistance backgrounds, leaf developmental stages, and plant ages, this research provides fundamental insights into the complex dynamics of agricultural plant pathogen coinfections.

## 1 Introduction

Plant diseases caused by different kinds of pathogens present a significant challenge to global food security, with coinfections in agricultural production systems being recognized increasingly as the norm rather than the exception (Tollenaere et al., 2016; Abdullah et al., 2017; Dutt et al., 2022; Priyadarshi et al., 2026). Pathogen coinfections can alter disease dynamics fundamentally through complex interactions that may increase, diminish, or have negligible effects on individual pathogen function (Seabloom et al., 2015; Abdullah et al., 2017; Kozanitas et al., 2017; Dutt et al., 2021). Despite this, the majority of plant disease research and plant breeding programs continue to focus on single-pathogen resistance which is likely limiting the effectiveness of their resistance approaches in real-world agricultural environments where pathogens co-occur on the same individual plant.

Canola (*Brassica napus*), which is the world’s second most important oilseed crop after soybean, suffers major production losses from viral and fungal pathogens (Zheng and Liu, 2022). Among the most economically important are *Leptosphaeria maculans*, the causative agent of blackleg disease, and turnip mosaic virus (TuMV), both of which can cause substantial yield losses independently (Fitt et al., 2006; Van de Wouw et al., 2016; Nellist et al., 2022; Abidin et al., 2026). These pathogens often occur together in canola-growing regions globally and consist of both non-RB and RB strains. Such strains pose ongoing challenges to achieving effective host-resistance-based management approaches (Balesdent et al., 2005; Guerret et al., 2017). Current canola cultivars incorporate various resistance mechanisms, including single dominant gene resistance (SDGR) and polygenic resistance (PGR), against *L. maculans* and TuMV (Walsh et al., 2002; Li et al., 2005; Marcroft et al., 2012; Raman et al., 2018; Nellist et al., 2022) and temperature-sensitive systemic invasion resistance (TSSIR) against TuMV (Nyalugwe et al., 2014; Abidin et al., 2025, Abidin et al., 2026).

Despite the importance of mixed pathogen infections, critical knowledge gaps persist regarding how they influence the efficacy of single-pathogen host resistances. Firstly, while most pathogen interaction research has focused on simultaneous infections or examined their effects on only one pathogen, knowledge is limited over how sequential infections affect resistance efficacy where one pathogen, e.g., a virus, has already become established systemically before secondary pathogen challenge occurs. Secondly, the role of plant developmental factors, especially leaf maturity and plant age, in mediating coinfection outcomes remains poorly understood despite evidence that these factors can influence individual pathogen–host interactions substantially (Moriones et al., 1998; Panter and Jones, 2002; Li et al., 2006b; Levy and Lapidot, 2008; Hull, 2014). Thirdly, comprehensive assessments of how coinfections influence both pathogens at the same time are rare, limiting our understanding of the reciprocal nature of pathogen interactions (Gutierrez et al., 2025).

There is a notable lack of research on viral and fungal coinfections in canola. Apart from preliminary observations over increased *L. maculans* disease severity in TuMV-infected leaves (Zarinjoo et al., 2020) and recent cotyledon-stage coinfection studies (Abidin et al., 2025), there is virtually no knowledge of how these two pathogens interact during different plant development stages or how such interactions impact the functionality of existing resistance mechanisms. This knowledge deficit is especially problematic because field infections typically involve multiple pathogen encounters involving different plant tissues and growth stages, conditions that may significantly undermine the robustness of resistance mechanisms developed under single-pathogen infection scenarios. Indeed understanding how pathogen coinfections influence resistance is crucial for developing sustainable disease management approaches. When coinfections compromise single-pathogen resistances, plant breeding programs may be selecting for traits that are likely to be of limited effectiveness in the field. Conversely, where the effectiveness of certain resistance mechanisms increases under coinfection conditions, this information could result in more robust host resistance strategies. Furthermore, the increasing prevalence of RB pathogen strains necessitates a deeper understanding of how pathogen interactions might accelerate or decelerate resistance breakdown in agricultural systems.

To address these knowledge gaps, we conducted a systematic investigation of virus–fungus coinfection dynamics in canola, focusing on the effects of leaf developmental stage, plant age, and different resistance mechanisms on pathogen interactions. Our study had three explicit objectives: (i) to determine how prior systemic TuMV infection affects subsequent *L. maculans* disease development in true leaves of varying maturity across plants of different ages of canola cultivars with different resistance mechanisms (SDGR, PGR, and TSSIR) or complete fungal susceptibility, (ii) to assess how secondary fungal infection with RB and non-RB *L. maculans* strains affects already established TuMV concentrations in leaves of different developmental stages on plants of different growth stages, and (iii) to evaluate whether the effectiveness of single-pathogen resistance mechanisms is maintained, enhanced, or compromised under sequential coinfection conditions, thereby providing insights for the development of improved integrated disease management strategies.

## 2 Materials and methods

### 2.1 Plant materials and growth conditions

Chinese cabbage (*B. rapa* subsp. *pekinensis*) cv. Wombok TuMV culture host and canola experimental plants were maintained in an air-conditioned glasshouse. The environmental regime was set at 22/18°C (day/night) with a 12-h photoperiod under natural light. The plants were grown in a standard potting mix within 9 × 9 × 15 cm square pots (one seed/pot), hand-watered daily, and fertilized weekly (Thrive, Yates Ltd., New South Wales, Australia). For the experiments, three canola cultivars, Scoop (with *L. maculans* PGR and TuMV TSSIR), Surpass 400 (with *L. maculans* SDGR), and Westar (without any *L. maculans* resistance) (Abidin et al., 2025), were used. Although the effectiveness of *L. maculans* SDGR and PGR has been demonstrated under typical field conditions (Marcroft et al., 2012: Sprague et al., 2006), this has not yet been done with TSSIR. TSSIR operates at 16 °C as no systemic TuMV invasion occurred at this temperature but breaks down at 28 °C (Nyalugwe et al., 2014). Under 22/18 °C (day/night) conditions, TSSIR is partially overcome in cv. Scoop when TuMV invades it systemically, but only reaches a low titer in systemically infected leaves (Abidin et al., 2026).

Westar always germinated 1 week earlier than the other two cultivars, so it was sown 7 days after Surpass 400 and Scoop to synchronize growth stages and ensure comparability across cultivars with different germination rates. Extra canola plants were grown for cotyledon virus mock inoculations (i.e., using healthy sap) and true leaf fungus mock inoculations (using deionized water) and to obtain cohorts of systemically virus-infected and virus-mock-inoculated plants with uniform leaf dimensions for subsequent *L. maculans* inoculation.

### 2.2 Virus isolate, inoculum preparation, and sap inoculation

**Figure 1:**
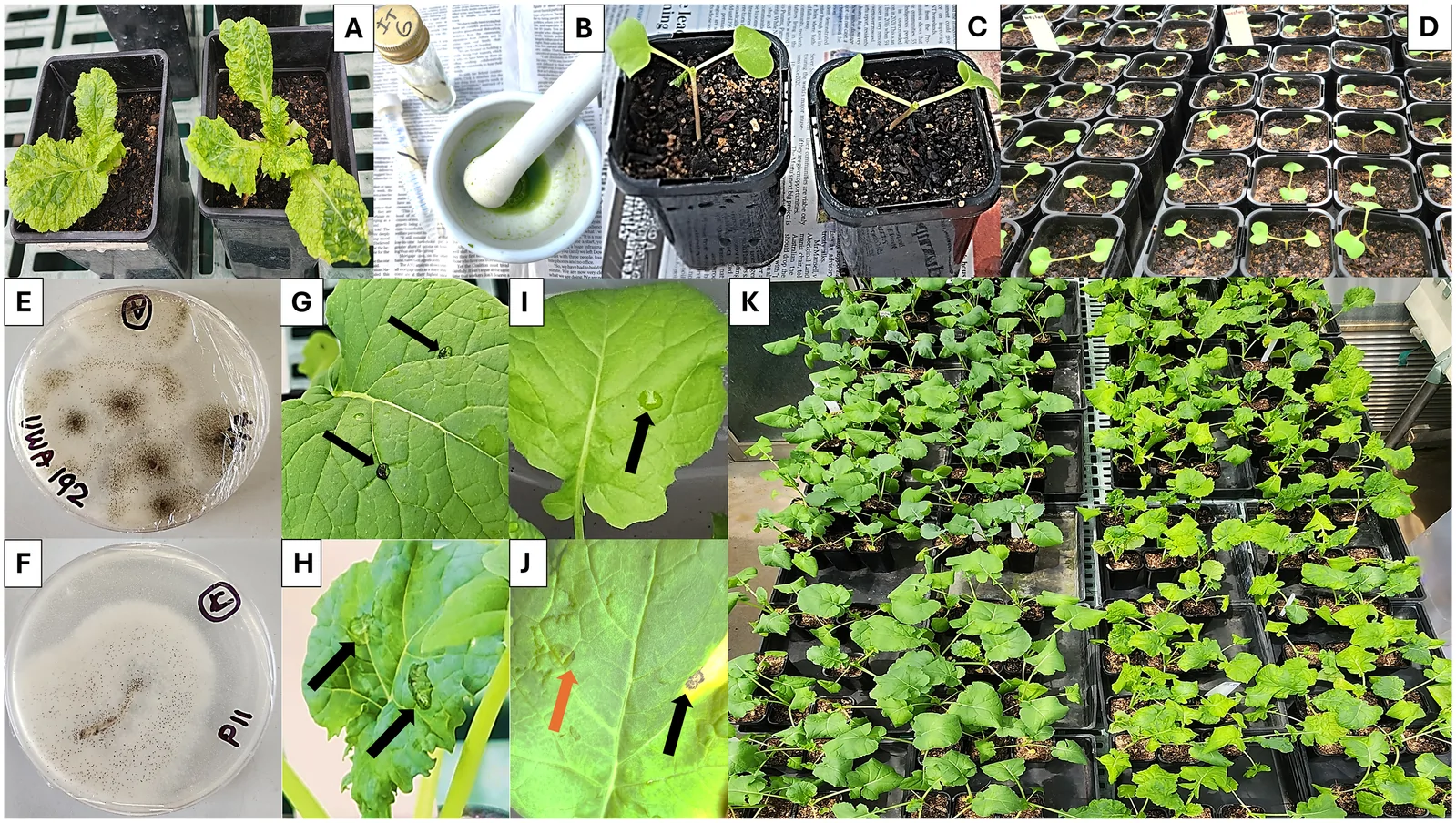
Explanatory methodology figure showing the different steps involved in the sequence of turnip mosaic virus (TuMV) sap, *Leptosphaeria maculans* drop, and both virus and fungus mock inoculations to canola (*Brassica napus*) seedlings and plants. The pathogen isolates used were 12.1 for the TuMV resistance breaking strain (RB) strain, UWA 192 for the *L. maculans* RB strain, and P11 for the *L. maculans* non-resistance breaking (non-RB) strain. Canola cotyledons were inoculated with sap from TuMV isolate 12.1-infected Chinese cabbage leaves 15–20 days after germination. Cotyledon virus mock inoculations with healthy sap were done immediately beforehand. The delay between cotyledon inoculation with TuMV and the first of the two *L. maculans* true leaf inoculations was 35 days at 1.05 SBGS and 50 days at 1.07 SBGS. The true leaves inoculated with *L. maculans* were at positions 2–5 for 1.05 SBGS and 2–7 for 1.07 SBGS (true leaf position leaf one excluded). The first *L. maculans* inoculation was to one half (right) of each true leaf followed by the second inoculation to its other half (left) either on the same day (0–0 dai) or 3 days later (0–3 dai). In these inoculations, a single drop (10 µL) of conidial inoculum (*L. maculans* inoculation) was applied to each half true leaf of the systemically TuMV-infected or virus-mock-inoculated plants. True leaf fungus mock inoculations with deionized water were done immediately beforehand. The three canola cultivars used had differing host resistances/susceptibilities, lacking any *L. maculans* resistances (Westar), *L. maculans* single dominant gene resistance (SDGR) (Surpass 400), and combined *L. maculans* polygenic resistance (PGR) and TuMV temperature-sensitive systemic invasion resistance (TSSIR) (Scoop). **(A)** TuMV isolate 12.1 culture in Chinese cabbage plants (infective sap source) showing leaf symptoms of severe mosaic, deformation, and size reduction with plant stunting. **(B)** Mortar and pestle was used to extract infective sap from TuMV-infected leaves before being mixed with Celite abrasive (in glass tube) and buffer solution (in plastic tube) before being rubbed onto canola cotyledons. **(C)** Two canola seedlings showing the growth stage when they were inoculated with TuMV-infected sap or mock-inoculated with healthy sap. **(D)** Arrangement of canola seedlings growing in small-sized pots when these sap inoculations were done. **(E)** Petri dish culture of *L. maculans* RB strain isolate UWA192 showing dark-colored hyphae and pycnidial production. **(F)** Petri dish culture *of L. maculans* non-RB strain isolate P11 showing lighter-colored hyphae and pycnidial production. **(G)** Two same-day drop inoculations with *L. maculans* isolate UWA192 onto each half of true leaf two systemically TuMV-infected (asymptomatically) on plant of cv. Scoop at growth stage 1.05 SBGS (black arrows indicate inoculation sites). **(H)** Two same-day drop inoculations with *L. maculans* isolate P11 onto each half of true leaf three systemically TuMV-infected (showing severe mosaic symptoms) on plant of cv. Westar at growth stage 1.05 SBGS (black arrows indicate inoculation sites). **(I)** Day 0 drop fungus mock inoculation (deionized water) onto right half of true leaf two on cotyledon virus-mock-inoculated plant (no symptoms) of cv. Surpass 400 at growth stage 1.07 SBGS (black arrow indicates inoculation site; left half still to be inoculated on day 3 (0–3 dai). **(J)** Day 0 and day 3 (0–3 dai) drop inoculations with *L. maculans* isolate UWA192 onto systemically TuMV-infected true leaf two (showing mild mosaic symptoms) on plant of cv. Surpass 400 at growth stage 1.07 SBGS; right half day 0 inoculation showing necrotic *L. maculans* symptoms (black arrow), left half day 3 (0–3 dai) inoculation (orange arrow), symptoms yet to appear. **(K)** Trays of canola cv. Westar plants at the 1.05 SBS plant growth stage when their true leaves 2–5 were drop-inoculated with *L. maculans* or fungus-mock-inoculated with deionized water (left, virus-mock-inoculated plants; right, systemically TuMV-infected plants showing chlorotic mosaic symptoms).

TuMV isolate 12.1 was used. It belongs to a RB strain that overcomes all known canola TuMV single gene resistances (Nyalugwe et al., 2014; Guerret et al., 2017; Jones et al., 2021) but fails to overcome canola TSSIR (Nyalugwe et al., 2014; Abidin et al., 2025, Abidin et al., 2026). This isolate was maintained via serial subculture in Chinese cabbage plants. For sap inoculations, infected leaf tissue (**Figure 1A**) was ground in a mortar and pestle using an extraction buffer consisting of 11.74 g L^-^¹ K_2_HPO_4_, 4.48 g L^-^¹ KH_2_PO_4_, and 1.26 g L^-^¹ Na_2_SO_3_ (**Figure 1B**). This extract was mixed with Celite abrasive and then rub-inoculated onto leaves of Chinese cabbage culture host plants, or cotyledons of healthy canola plants for the experiments (**Figures 1C, D**). For virus mock inoculations, leaf extracts from healthy Chinese cabbage plants mixed with Celite were inoculated onto canola cotyledons.

### 2.3 Fungal isolates, inoculum preparation, and inoculation

Two *L. maculans* isolates were used: RB strain isolate UWA192 (overcomes *LepR3*/SDGR in cv. Surpass 400) and non-RB strain isolate P11. Conidial suspensions were prepared according to Li et al. (2003) and Abidin et al. (2025). Lyophilized cultures were grown on V8 juice agar (159 mL V8 juice, 1.5 g CaCO_3_, 15 g agar, and 1 L deionized water) and incubated at 22 °C under fluorescent and UV light for 7 days on agar plates until mature pycnidia were visible (**Figures 1E, F**). Conidia were harvested in deionized water filtered through Mira cloth (Calbiochem, Perth, Australia) and adjusted to 10^7^ spores mL^-1^ using a hemocytometer counting chamber. A 10-μL droplet of this conidial inoculum was applied to the right half and another to the left half of each already systemically TuMV-infected true leaf or similarly of the true leaves of virus-mock-inoculated plants (**Figures 1G–J**). The fungus-mock-inoculated leaves received deionized water droplet inoculum instead (**Figure 1I**). **Figure 1K** shows the growth stage at which true leaves 2–7 of the canola plants were droplet-inoculated and their arrangement in trays at 1.07 SBGS (left-virus-mock-inoculated, right-systemically TuMV infected). After droplet inoculation, canola plants were incubated inside clear plastic boxes for 96 h to maintain a high-humidity environment (>90% RH) conducive to typical *L. maculans* disease lesion development.

### 2.4 Virus detection

Tip leaves from canola plants were collected and placed into small resealable bags and put in cooler boxes with ice packs. TuMV presence was determined by triple-antibody sandwich Enzyme Linked Immunosorbent Assay (TAS-ELISA) using Agdia Pathoscreen Kits (Agdia Inc., Elkhart, Indiana, USA) following the manufacturer’s protocol. Sample extracts were placed in Agdia’s 96-well microtiter plates, each sample being loaded into a paired well, including positive and negative controls containing sap extracts from TuMV-infected or healthy leaf samples, respectively. A Bio-Rad microplate reader (model 680; Bio-Sys) was used to measure optical density (OD) absorbance values at 405 nm (A_405_). Sample A_405_ values of two or more times those of leaf sample extracts from healthy control plants were considered TuMV-positive.

### 2.5 Inoculation sequences and timing

Canola cotyledons were inoculated with sap from TuMV isolate 12.1-infected Chinese cabbage leaves at 15–20 days after germination, by which time they had just developed their first very small true leaf. Virus mock inoculations to cotyledons of control plants were performed just before these TuMV inoculations. At 30 days after cotyledon inoculation (dai), true leaves were assessed for systemic TuMV symptoms and also sampled for testing using ELISA. This was done to establish the viral symptom intensity (SI) and virus concentration (VC) values of systemically infected leaves prior to *L. maculans* inoculation and ensure the virus-free status of virus-mock-inoculated plants. The procedures used to obtain these two values are described in Section 2.6.

The TuMV-inoculated and virus-mock-inoculated canola plants were grown either to the fifth true leaf stage [i.e., cotyledon, first (i.e., lowest) to fifth (i.e., topmost) leaves] or the seventh true leaf stage [i.e., cotyledon, first (lowest) to 7th (topmost) leaves]. The former corresponds with the 1.05 and the latter with the 1.07 Sylvester–Bradley growth stages (SBGS) (Sylvester-Bradley et al., 1984). These growth stages were selected in a previous study (Li et al., 2006b) in which cv. Surpass 400 and cv. Westar developed their highest *L. maculans* disease severities when inoculated at 1.05 or 1.07 SBGS, respectively. Once the plants had reached growth stages of 1.05 and 1.07 SBGS, the first *L. maculans* inoculation was to one half (right) of each true leaf followed by the second inoculation to its other half (left) either on the same day (0–0 dai) or 3 days later (0–3 dai) (**Table 1**; **Figures 1G–K**). These two specific double-inoculation sequences (i.e., both on the same day or the second delayed by 3 days) were used previously by Abidin et al. (2025), so adopting them here could facilitate a comparison of the research findings. The true leaves inoculated were in positions 2–5 or 2–7 at 1.05 or 1.07 SBGS, respectively. The interval between cotyledon TuMV inoculation and the first *L. maculans* true leaf inoculation was 35 days at 1.05 SBGS and 50 days at 1.07 SBGS. Control fungus mock inoculations to true leaves of control plants were performed just before these *L. maculans* inoculations.

**Table 1:** Experimental treatments involving initial virus and subsequent fungus infection.

Treatment	Virus-inoculated or virus-mock-inoculated	Fungus-inoculated or fungus-mock-inoculated	Same day (0–0 dai) or delayed (0–3 dai) 2nd fungal inoculation
1	Mock-inoculated	Deionized water	0–0
2	Mock-inoculated	Non-RB (first)–non-RB (second)	0–0
3	Mock-inoculated	Non-RB (first)–RB (second)	0–0
4	Mock-inoculated	RB (first)–non-RB (second)	0–0
5	Mock-inoculated	RB (first)–RB (second)	0–0
6	Virus-infected	Deionized water	0–0
7	Virus-infected	Non-RB (first)–non-RB (second)	0–0
8	Virus-infected	Non-RB (first)–RB (second)	0–0
9	Virus-infected	RB (first)–non-RB (second)	0–0
10	Virus-infected	RB (first)–RB (second)	0–0
11	Mock-inoculated	Deionized water	0–3
12	Mock-inoculated	Non-RB (first)–non-RB (second)	0–3
13	Mock-inoculated	Non-RB (first)–RB (second)	0–3
14	Mock-inoculated	RB (first)–non-RB (second)	0–3
15	Mock-inoculated	RB (first)–RB (second)	0–3
16	Virus-infected	Deionized water	0–3
17	Virus-infected	Non-RB (first)–non-RB (second)	0–3
18	Virus-infected	Non-RB (first)–RB (second)	0–3
19	Virus-infected	RB (first)–non-RB (second)	0–3
20	Virus-infected	RB (first)–RB (second)	0–3

Initial canola (*Brassica napus*) cotyledon inoculation with turnip mosaic virus (TuMV) resistance breaking (RB) strain was followed by two subsequent true leaf inoculations with *Leptosphaeria maculans* non-resistance breaking (non-RB) and/or RB strains. The pathogen isolates used were 12.1 for the TuMV RB strain, UWA 192 for *L. maculans* RB strain, and P11 for *L. maculans* non-RB strain. The delay between initial cotyledon inoculation with TuMV and *L. maculans* true leaf inoculation was 35 days at the 1.05 Sylvester–Bradley growth stage (SBGS) and 50 days at 1.07 SBGS. Second *L. maculans* inoculations were either on the same day as the first (=0–0 dai) or 3 days afterward (=0–3 dai) (i.e., first inoculation at day 0 and second inoculation at day 3). Mock-inoculated means cotyledons inoculated with healthy plant sap. Deonized water was used for fungus mock inoculations.

### 2.6 Assessments for fungal and virus disease severity and virus concentration

*L. maculans* symptoms on leaves 2–5 at 1.05 SBGS and leaves 2–7 at 1.07 SBGS were recorded 11 days after the second *L. maculans* inoculation for the 0–0 dai assessment and 14 days after the 0–3 dai assessment. The symptoms were scored based on a progressive qualitative 0–5 scale (McGee and Petrie, 1978; Williams, 1985), where 0 = no visible symptoms, 1 = necrotic hypersensitive (localized tiny dark brown or black necrotic spots), 2 = gray-green tissue collapsed spots (2 to 3 mm in diameter) with diffuse margins, 3 = collapsed gray-green tissue (>3 mm in diameter) with diffuse margins, 4 = collapsed gray-green tissue spots and diffuse margin with a few pycnidia, and 5 = collapsed gray-green tissue with masses of pycnidia/plant death. Maximum disease severity that effectively hit a saturation point was reached within the *L. maculans* lesion boundary on each inoculated half cotyledon of cv. Westar inoculated at 10^7^ spores mL^-^¹. *L. maculans* percent leaf disease index (%LDI) values were based on a severity score from a 0–5 scale modified from Murtza et al. (2022). Each 0–5 diseased leaf score was calculated as follows: %LDI = ([(a × 0) + (b × 1) + (c × 2) + (d × 3) + (e × 4) + (f × 5)] × 100)/[(a + b + c + d + e + f) × 5)], where a–f are the numbers of plants for each disease score category.

Viral SI was assessed using a simplified version of the of rating scale of McKirdy et al. (2000), where 0 = asymptomatic, 1 = mild mosaic, 2 = moderate mosaic, 3 = severe mosaic, 4 = very severe mosaic, and 5 = plant death. Estimates of VC were derived from antigen accumulation obtained from exponentially (*e^x^*) transformed A_405_ values (Wilson and Jones, 1993). The baseline SI and initial VC assessments preceded the first *L. maculans* inoculation by 5 days, both being made 30 days after cotyledon TuMV inoculation. The final VC assessment was delayed until 12 to 13 days after the second *L. maculans* inoculation.

### 2.7 Experimental design and statistical analysis

Each experiment was fully duplicated and followed a factorial design: two plant growth stages (i.e., 1.05 and 1.07 SBGS) × three canola cultivars (Surpass 400, Scoop, and Westar) × two virus (virus-infected and virus-mock-inoculated) × five fungal treatments (four fungal strain combinations and a fungus-mock-inoculated control) × two fungal inoculation timings (0 and 3 dai) (**Table 1**). There were eight replications per treatment, and each experiment comprised 960 pots. Before being analyzed, the %LDI and *e^x^*-transformed VC values were tested for normality and homogeneity of variance which they fully satisfied. To ensure that the mathematical assumptions of GenStat’s 23rd edition (Lawes Agricultural Trust, Rothamsted Research, UK) analysis of variance (ANOVA) were preserved, these tests were performed after *e^x^* transformation of A_405_ VC values.

*T*-tests and *F*-tests confirmed that there were no significant differences (*P* > 0.05) between the original and repeat experiments. Consequently, the two data sets were combined before analysis as a single data set. ANOVA was performed using GenStat with Fisher’s least significant differences (LSDs) at *P* < 0.05 used to identify significant differences between treatment means. Since Tukey’s honestly significant difference (HSD) increases type II errors (i.e., failure to detect actual differences), Fisher’s LSD was used instead in our factorial analyses because it maximizes statistical power to detect subtle effects (Carmer and Swanson, 1973; Maxwell et al., 2017; Montgomery, 2019). Because adopting this approach with a large factorial design can inflate the family-wise error rate (FWER) and increase the probability of false positives, to mitigate this potential drawback we utilized an *F*-test which provides standard protection against FWER inflation prior to LSD testing.

**Figure 2:**
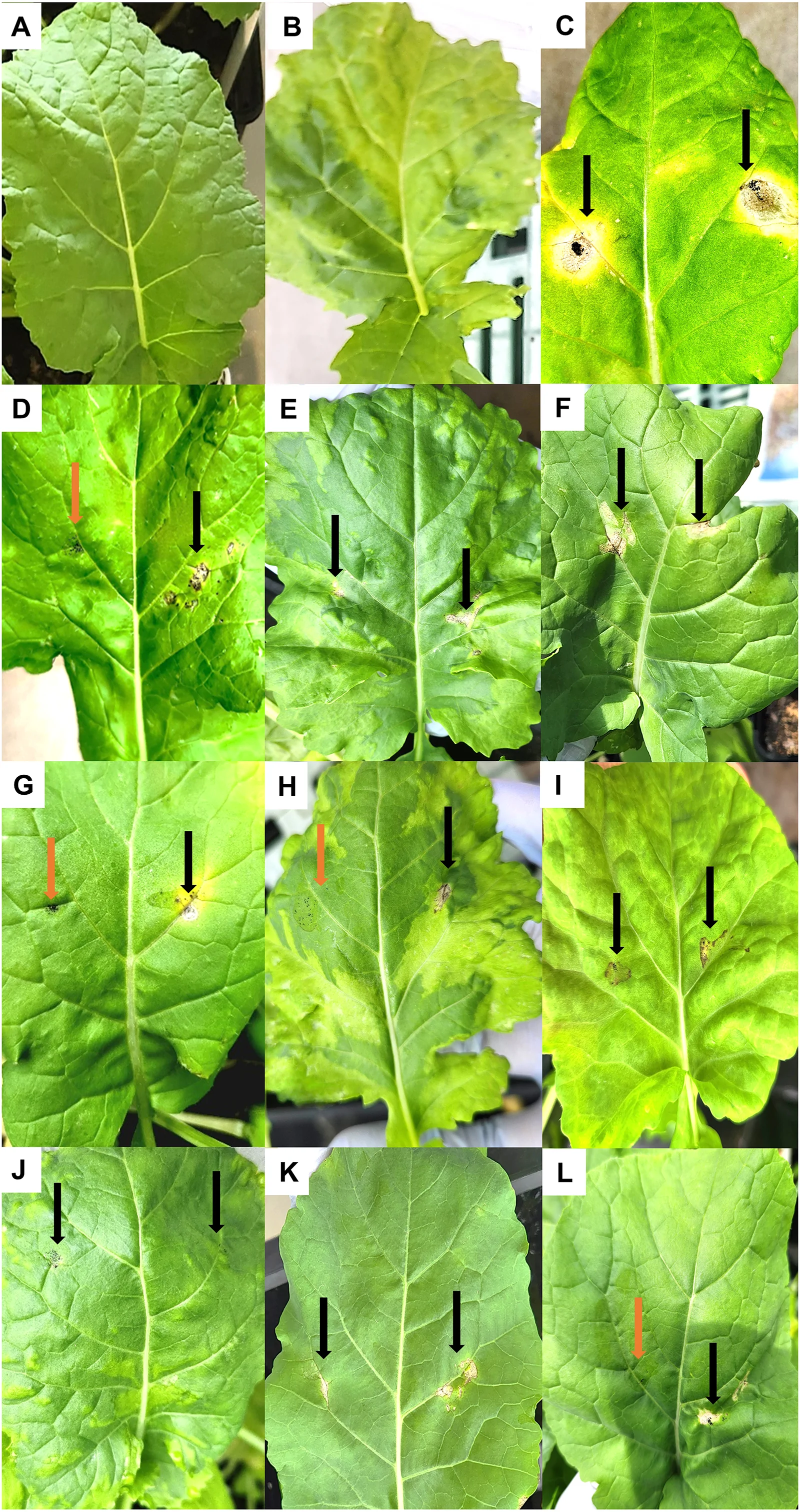
Drop inoculation of *Leptosphaeria maculans* or fungus-mock-inoculated with deionized water to systemically turnip mosaic virus (TuMV)-infected or healthy true leaves following infective TuMV sap inoculation or virus mock-inoculation to cotyledons of three canola (*Brassica napus*) cultivars with differing host resistances: lacking *L. maculans* resistance (Westar), *L. maculans* single dominant gene resistance (SDGR) (Surpass 400), and combined *L. maculans* polygenic resistance (PGR) and TuMV temperature-sensitive systemic invasion resistance (TSSIR) (Scoop). These drop inoculations to canola leaves were made at Sylvester-Bradley growth stages (SBGS) 1.05 (i.e., younger plants) or 1.07 (i.e., older plants). Leaf positions refer to leaf numbering starting at the second oldest true leaf (leaf two) and ending at the seventh (i.e., youngest) leaf above it (leaf seven). Leaf from cotyledon virus-mock-inoculated plant with fungus mock inoculation **(A)**. Leaf from cotyledon TuMV-inoculated plant with fungus mock inoculation **(B)**. Symptoms following *L. maculans* drop inoculation on cultivars Westar **(C–E)**, Surpass 400 **(F–I)**, and Scoop **(J–L)**. Two black paired arrows on each half-leaf in the same image indicate *L. maculans* symptoms following the first *L. maculans* inoculation (right half) and the second *L. maculans* inoculation on same day (0–0 dai; left half). Single black (right) and orange (left) paired arrows on each half-leaf in the same image indicate the first *L. maculans* inoculation on day 0 and the second *L. maculans* inoculation 3 days afterward (0–3 dai). **(A)** cv. Scoop leaf position 2 at 1.07 SBGS both same-day inoculations (both deionized water inoculations, no symptoms). **(B)** cv. Surpass 400 leaf position 2 at 1.07 SBGS first inoculation on day 0 and second inoculation on day 3 (0–3 dai) (both deionized water inoculations, no necrotic lesions, mosaic). **(C)** cv. Westar initially cotyledon virus-mock-inoculated and subsequently leaf position 2 inoculated with *L. maculans* isolates P11 (first) and UWA192 (second) both on day 0 at 1.05 SBGS (large necrotic lesions). **(D)** cv. Westar initially cotyledon virus-mock-inoculated and subsequently leaf position 3 inoculated with *L. maculans* isolates P11 (first) at day 0 followed by P11 (second) 3 days afterward (0–3 dai) at 1.05 SBGS (smaller necrotic lesion on day 3). **(E)** cv. Westar initially cotyledon TuMV-inoculated and subsequently leaf position 3 inoculated with *L. maculans* isolates P11 (first) and P11 (second), both on day 0 at 1.07 SBGS (necrotic lesion sizes both decreased by TuMV coinfection, severe mosaic). **(F)** cv. Surpass 400 initially cotyledon virus-mock-inoculated and subsequently leaf position 2 inoculated with *L. maculans* isolates UWA192 (first) and UWA 192 (second) both on day 0 at 1.07 SBGS (necrotic lesions). **(G)** cv. Surpass 400 initially cotyledon virus-mock-inoculated and subsequently leaf position 3 inoculated with *L. maculans* isolates UWA192 on day 0 followed by P11–3 days afterward (0–3 dai) at 1.05 SBGS (smaller necrotic lesion on day 3). **(H)** cv. Surpass 400 initially cotyledon TuMV-inoculated and subsequently leaf position 3 inoculated with *L. maculans* isolates UWA192 (first) on day 0 followed by P11 (second) on day 3 (0–3 dai) at 1.05 SBGS (necrotic lesion sizes both decreased by TuMV coinfection, smaller necrotic lesions on day 3, severe mosaic). **(I)** cv. Surpass 400 initially cotyledon TuMV-inoculated and subsequently leaf position 2 inoculated with *L. maculans* isolates UWA192 (first) and UWA192 (second) both on day 0 at 1.07 SBGS (necrotic lesion sizes both decreased by TuMV coinfection, mosaic). **(J)** cv. Scoop initially cotyledon TuMV-inoculated and subsequently leaf position 2 inoculated with *L. maculans* isolates P11 (first) on day 0 and P11 (second) on day 3 (0–3 dai) at 1.05 SBGS (no necrotic lesions, mild mosaic). **(K)** cv. Scoop initially cotyledon virus-mock-inoculated and subsequently leaf position 2 inoculated with *L. maculans* isolate UWA192 (first) and UWA192 (second) both on day 0 at 1.07 SBGS (medium-sized necrotic lesions). **(L)** cv. Scoop initially cotyledon virus-mock-inoculated and subsequently leaf position 3 inoculated with *L. maculans* isolates UWA192 (first) on day 0 and P11 (second) on day 3 (0–3 dai) at 1.07 SBGS (day 3 no necrotic lesion).

No virus disease symptoms ever developed in any cotyledon virus-mock-inoculated plants (**Figure 2A**) or *L. maculans* disease symptoms in any true leaf fungus-mock-inoculated plants (**Figure 2B**). As fungus-mock-inoculated plants always developed 0%LDI values and virus-mock-inoculated plants always delivered 1.1 VC values, they were both excluded from the statistical analysis for %LDI or VC values, respectively, to prevent any data bias.

## 3 Results

### 3.1 Statistical significance of main effects and interactions for fungal leaf disease

At 11 days after the second fungal inoculation, statistically significant main effects for *L. maculans* %LDI (all *P* < 0.001), unless otherwise indicated, were obtained across all factors (**Supplementary Table 1**): canola cultivar, virus status (virus-infected, virus-mock-inoculated, or fungus-mock-inoculated plants), leaf position (2–5 at 1.05 SBGS and 2–7 at 1.07 SBGS), second fungal inoculation times (0 or 3 dai), and fungal non-RB and RB strain combinations. All possible two-way interactions for %LDI at growth stages 1.05 and to 1.07 SBGS were highly significant (*P* < 0.001). The only exceptions were the 1.05 SBGS %LDI values for virus presence × inoculation time and the 1.07 SBGS %LDI values for fungal strain combination × inoculation time, both of which were significant at *P* < 0.05. Regarding the three-way interactions, the %LDI values were highly significant (*P* < 0.001) at SBGS growth stages 1.05 and 1.07 for cultivar × virus × fungus, cultivar × virus × leaf position, cultivar × fungus × leaf position, cultivar × virus × inoculation time, and cultivar × leaf position × inoculation time. The interaction cultivar × fungus × inoculation time was significant for both growth stages at *P* < 0.05. Additionally, three-way interactions (*P* < 0.05) were present at growth stage 1.05 SBGS for virus × fungus × inoculation time and for 1.07 SBGS for virus × leaf position × inoculation time.

### 3.2 Statistical significance of main effects and interactions for virus concentration

At 12 to 13 days after the second fungal inoculation to the three canola cultivars, there were statistically significant (all *P* < 0.001) main effects for virus VC expressed as *e^x^* transformed A_405_ values in relation to different canola cultivars, leaf positions (1.05 SBGS leaf positions 2–5; 1.07 SBGS, leaf positions 2–7), second fungus inoculation times (0–0 or 0–3 dai), and fungus strains/isolates (non-RB P11 or RB UWA192). All possible two- and three-way interactions in relation to both 1.05 and to 1.07 SBGS *e^x^* transformed A_405_ values were significant at *P* < 0.001.

### 3.3 Virus symptom appearance and concentration before fungal inoculation

**Figure 3:**
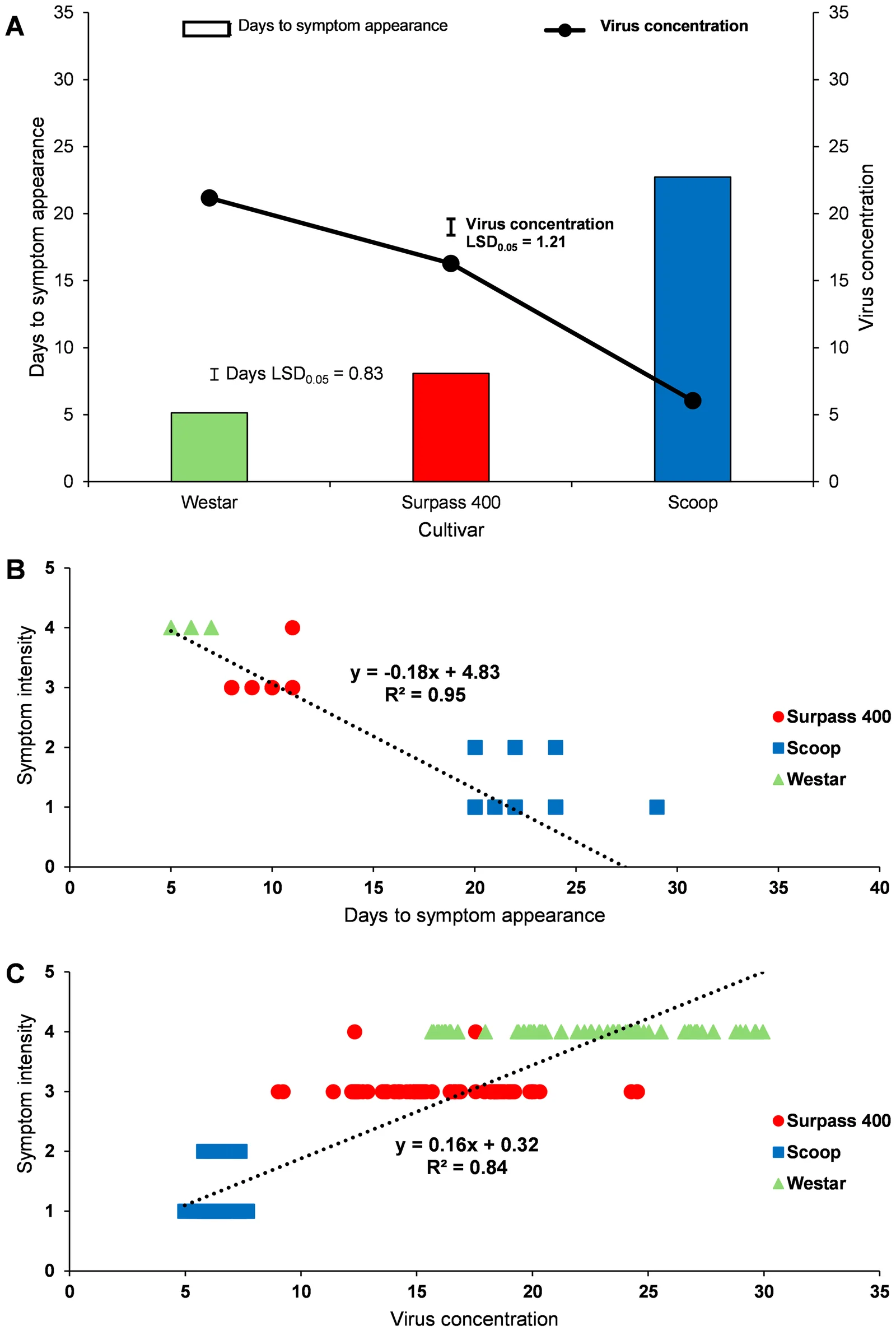
**(A)** Bar and line chart showing the average number of days to first turnip mosaic virus (TuMV) systemic symptom appearance (left axis) and virus concentration (VC) (right axis) for systemically TuMV-infected plants of three canola (*Brassica napus*) cultivars. VC is expressed as ELISA optical density exponentially (*e^x^*) transformed A_405_ values from tip leaf samples collected 30 days after TuMV cotyledon inoculation. LSD bar (at *P* < 0.05) represents significant differences between cultivars (based on cultivar means). **(B)** Relationship between TuMV symptom intensity (SI) values and the number of days to first TuMV systemic symptom appearance in the three cultivars in young leaves 30 days after TuMV cotyledon inoculation (based on individual replicate data). Symptom intensity expressed according to 0–5 symptom severity scale values (0 = asymptomatic to 5 = plant death). **(C)** Relationship between TuMV SI and VC values in young leaves at 30 days after TuMV cotyledon inoculation. Cultivar resistance/susceptibility status: without *L. maculans* resistance (Westar), *L. maculans* single dominant gene resistance (SDGR) (Surpass 400), and combined *L. maculans* polygenic resistance (PGR) and TuMV temperature-sensitive systemic invasion resistance (TSSIR) (Scoop) (based on individual replicate data).

After cotyledon inoculation, systemic symptoms appeared fastest in Westar (at 5.1 dai), followed soon afterward in Surpass 400 (at 8.1 dai), but much later in Scoop (at 22.7 dai) (**Figure 3A**). ELISA tests on tip leaf samples collected at 30 dai revealed significant differences (*P* < 0.001) in VC values. These were highest in Westar (21.2) and intermediate in Surpass 400 (16.3) but much lower in Scoop (6.1) (**Figure 3A**). Similarly, at 30 dai, the SI values were highest in Westar and intermediate in Surpass 400 but lowest in Scoop. They were strongly negatively correlated (*R*^2^ = 0.95; **Figure 3B**) with time of first symptom appearance but strongly positively correlated with VC values (*R*^2^ = 0.84; **Figure 3C**). Therefore, the TSSIR presence in Scoop not only delayed virus symptom onset but also significantly diminished both SI and VC values. The direct relationship between SI and VC values found here aligns with earlier findings with other viruses, such as potato leaf roll virus (genus *Polerovirus*, family *Solemoviridae*) in potato (*Solanum tuberosum*) (Wilson and Jones, 1993).

### 3.4 Effect on cultivars with different resistance characteristics

**Figure 4:**
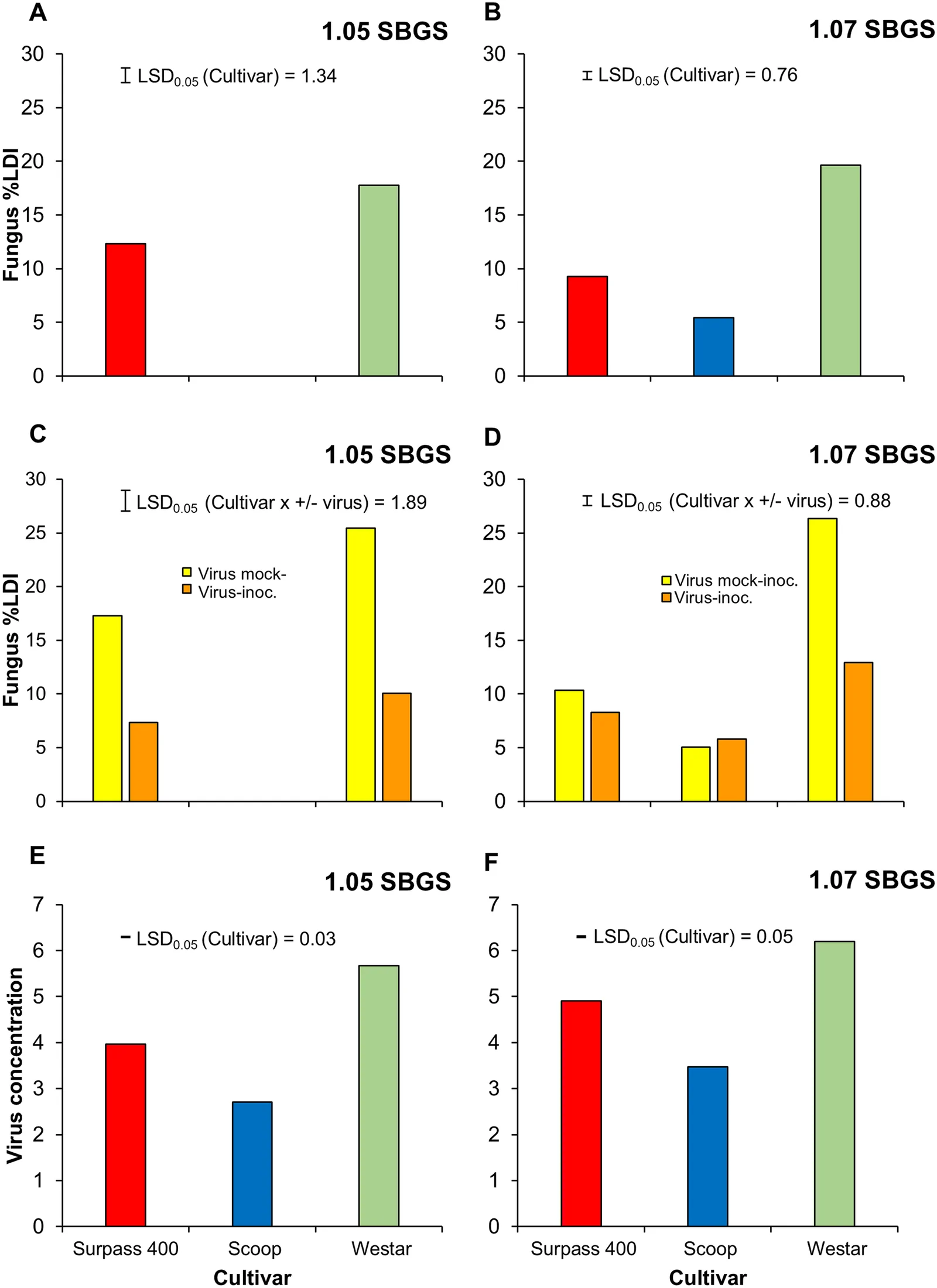
*Leptosphaeria maculans* percentage leaf disease index (%LDI) and turnip mosaic virus (TuMV) concentration (ELISA optical density exponentially (*e^x^*) transformed A_405_ virus concentration (VC) values in true leaves of systemically TuMV-infected or without TuMV infection growing on plants of three canola (*Brassica napus*) cultivars. **(A)** *L. maculans* combined %LDI values from leaf positions 2–5 at 1.05 SBGS. **(B)** *L. maculans* combined %LDI values from leaf positions 2–7 at 1.07 SBGS. **(C)** *L. maculans* %LDI values from leaves without TuMV infection (i.e., from cotyledon mock-inoculated plants) or with TuMV infection (i.e., systemically virus-infected) for leaf positions 2–5 at 1.05 SBGS. **(D)** *L. maculans* %LDI values from leaves without or with TuMV infection for leaf positions 2–7 at 1.07 SBGS. **(E)** VC combined values from leaf positions 2–5 at 1.05 SBGS. **(F)** VC combined values from leaf positions 2–7 at 1.07 SBGS. LSD bars represent significant differences at *P* < 0.05. Mock-inoc., cotyledon mock-inoculated with healthy sap; Virus-inoc., cotyledon TuMV-inoculated leading to systemic virus infection. Cultivar resistance/susceptibility status: without *L. maculans* resistance (Westar), *L. maculans* single dominant gene resistance (SDGR) (Surpass 400), and combined *L. maculans* polygenic resistance (PGR) and TuMV temperature-sensitive invasion resistance (TSSIR) (Scoop). To obtain true leaves with or without systemic TuMV infection, cotyledons had previously been inoculated with TuMV-infective sap or virus-mock-inoculated with healthy sap, respectively. The true leaves inoculated with *L. maculans* were at positions 2–5 (at 1.05 Sylvester-Bradley growth stage (SBGS) or 2–7 at 1.07 SBGS). The delay between cotyledon inoculation with TuMV and the first of the two *L. maculans* true leaf inoculations was 35 days at 1.05 SBGS and 50 days at 1.07 SBGS. *L. maculans* drop inoculations were performed on different halves of each true leaf, the first inoculation on day 0 and the second at either of two inoculation timings (0–0 and 0–3 dai, respectively). *L. maculans* %LDI values were recorded on each half-leaf 11 days after the second *L. maculans* inoculations. VC values were from true leaves sampled 12 to 13 days after the second *L. maculans* inoculation.

Significant differences between cultivars (*P* < 0.001) were found for both %LDI and VC values within true leaves at growth stages 1.05 and 1.07 SBGS (**Figures 4A–F**). A consistent %LDI pattern emerged across both growth stages: from highest values across both 1.05 and at 1.07 SBGS for cultivar Westar without fungal resistance (17.8 and 19.6; **Figures 4A, B**), which were significantly higher than those of the cultivar Surpass 400 with SDGR (12.3 and 9.3) and the cultivar Scoop with PGR+TSSIR (0 and 5.4). At 1.05 SBGS, virus infection reduced %LDI to a greater extent compared to the higher values in mock-inoculated plants in both the cultivar without fungal resistances (10.1 vs. 25.4) or the cultivar with fungal SDGR 400 (7.4 vs. 17.3) (**Figure 4C**). This trend continued at 1.07 SBGS for the cultivar without fungal resistances (12.9 vs. 26.3) and the cultivar with SDGR (8.3 vs. 10.3) (**Figure 4D**). Conversely, the cultivar with PGR+TSSIR always maintained 0%LDI values (=complete resistance) at 1.05 SBGS, with only a slight increase at 1.07 SBGS (5.0–5.8; **Figure 4D**) (=partial resistance). In terms of VC, as with %LDI, the cultivar without resistance developed the highest values (5.7 and 6.1 at 1.05 and 1.07 SBGS, respectively; **Figures 4E,F**), and the cultivar with SDGR developed significantly lower VC values (4.0 and 4.9 at 1.05 and 1.07 SBGS, respectively) (**Figure 4E**), followed by the cultivar with PGR+TSSIR with even lower VC values of 2.7 and 3.5, respectively, at 1.05 and 1.07 SBGS (**Figure 4F**). Thus, the combination of fungal PGR and virus TSSIR conferred full fungal resistance at 1.05 SBGS and partial fungal resistance at 1.07 SBGS. Moreover, while fungal SDGR suppressed %LDI, its efficacy at doing this was greater when the virus was absent. Additionally, the greatest decrease in VC values occurred when PGR and TSSIR were both present.

### 3.5 Effect of leaf maturity and second inoculation time upon fungal disease

**Figure 5:**
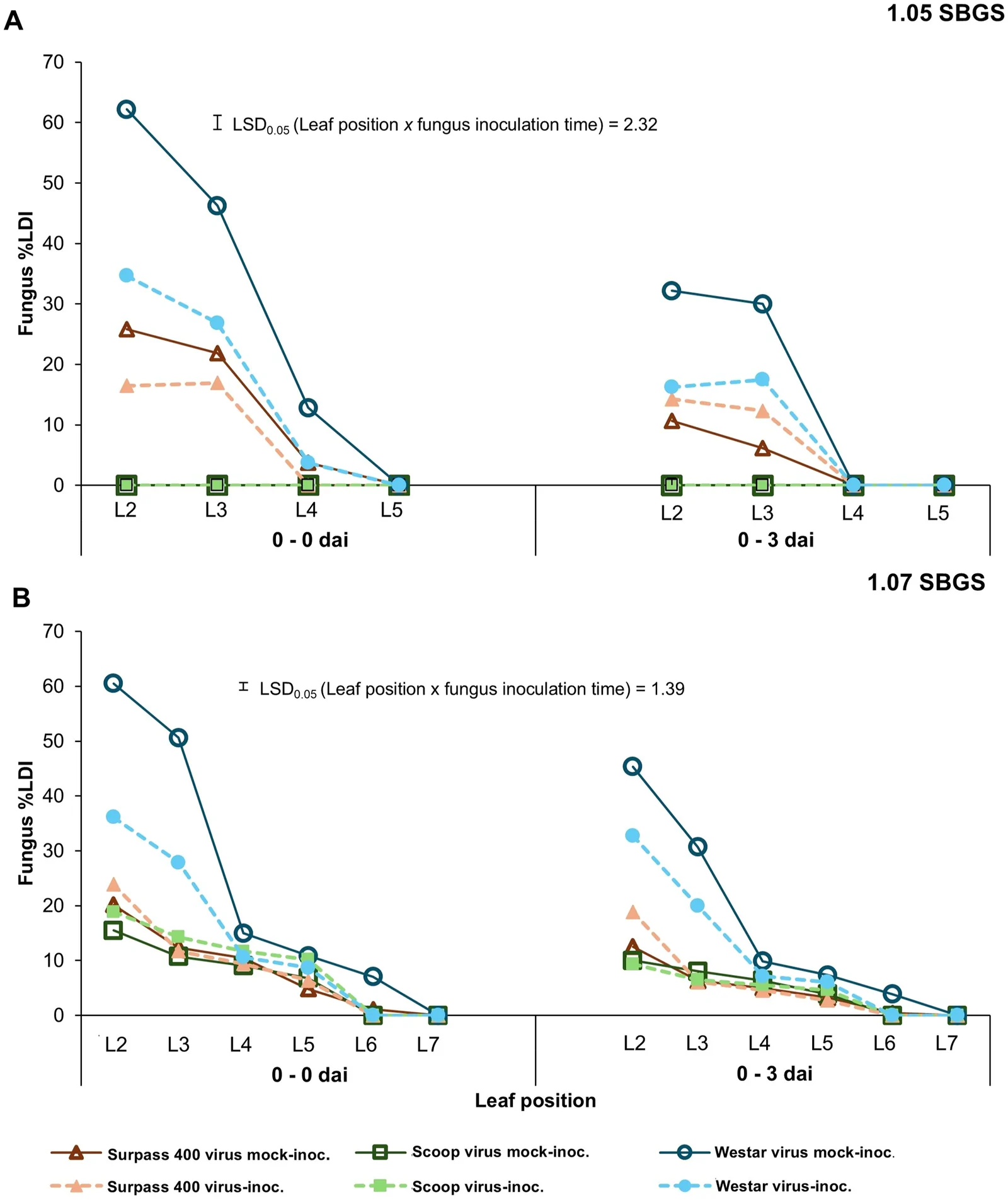
*Leptosphaeria maculans* percentage leaf disease index (%LDI) values on true leaves systemically Turnip mosaic virus (TuMV) infected or lacking TuMV infection growing on plants of three canola (*Brassica napus*) cultivars. **(A)** Individual *L. maculans* %LDI values for each of leaf positions 2–5 at second inoculation times of 0–0 and 0–3 dai for leaves with or without TuMV infection of each cultivar at growth stage 1.05 SBGS. **(B)** Individual *L. maculans* %LDI values for each of leaf positions 2–7 at 0–0 and 0–3 dai for leaves with or without TuMV infection of each cultivar at 1.07 SBGS. LSD bars represent significant differences at *P* < 0.05 between different leaf position and *L. maculans* second inoculation time. Mock-inoc., cotyledon virus-mock-inoculated; Virus-inoc., cotyledon TuMV-inoculated leading to systemic virus infection. Cultivar resistance/susceptibility status: without *L. maculans* resistance (Westar), *L. maculans* single dominant gene resistance (SDGR) (Surpass 400), and combined *L. maculans* polygenic resistance (PGR) and TuMV temperature-sensitive invasion resistance (TSSIR) (Scoop). To obtain true leaves with or without TuMV infection, cotyledons had previously been inoculated with TuMV-infective sap or virus-mock-inoculated with healthy sap, respectively. The true leaves inoculated with *L. maculans* were at positions 2–5 (at 1.05 Sylvester–Bradley growth stage (SBGS) or 2–7 (at 1.07 SBGS). The delay between cotyledon inoculation with TuMV and the first of the two *L. maculans* true leaf inoculations was 35 days at 1.05 SBGS and 50 days at 1.07 SBGS. *L. maculans* drop inoculations were performed on different halves of each true leaf, the first inoculation on day 0 and the second at either of two *L. maculans* inoculation timings (0–0 and 0–3 dai, respectively). *L. maculans* %LDI values were recorded on each half-leaf 11 days after the second *L. maculans* inoculation.

At both 1.05 and 1.07 SBGS, there were significant (*P* < 0.001) fungal %LDI differences in terms of both leaf age and position and inoculation time. Except with cv. Scoop at 1.05 SBGS, the overall %LDI values showed a similar trend of decreasing symptom severity across both inoculation times (0–0 and 0–3 dai) as the leaf position upward on the stem increased, with the oldest bottom leaves always developing the highest values whereas the youngest upper leaves never developed any. This trend was evident at both 1.05 SBGS (for leaves 2 to 5) and 1.07 SBGS (for leaves 2 to 7) for both inoculation times (0–0 and 0–3 dai) (**Figures 5A, B**). For cv. Westar, virus mock inoculation to cotyledons resulted in the highest %LDI values on true leaves 2 and 3 at 1.05 SBGS for 0–0 dai (62.2 and 46.3, respectively) and 0–3 dai (32.2 and 30.0, respectively) (**Figure 2C, D**, **5A**) and at 1.07 SBGS for 0–0 dai (60.5 and 50.6, respectively) and 0–3 dai (45.4 and 30.7, respectively) (**Figure 5B**). Systemically virus-infected Westar produced the second highest values for leaves 2 and 3 at 1.05 SBGS for 0–0 dai (34.7 and 26.9, respectively) and for 0–3 dai (16.3 and 17.5, respectively) (**Figure 5A**) and at 1.07 SBGS for 0–0 dai (36.1 and 27.9, respectively) and 0–3 dai (32.8 and 19.9, respectively) (**Figures 2E, B**). With Surpass 400, for 1.05 SBGS at 0–0 dai, virus mock inoculation resulted in significantly higher %LDI values (25.8 for leaf 2 and 21.2 for leaf 3) as compared with systemically virus-infected plants (16.5 for leaf 2 and 16.9 for leaf 3) (**Figure 5A**). In contrast, for Surpass 400 for 1.05 SBGS at 0–3 dai, systemically virus-infected plants developed higher %LDI values than virus-mock-inoculated plants for leaf 2 (14.3 versus 10.7) and leaf 3 (12.3 versus 6.19) (**Figures 2G, H**, **5A**). For 1.07 SBGS, at 0–0 dai, leaf 2 had a higher %LDI value for systemically virus-infected plants (23.9) than for virus-mock-inoculated plants (20.2), but at 0–3 dai the corresponding %LDI values for systemically virus-infected versus virus-mock-inoculated plants differed more (18.9 vs. 12.6) (**Figures 2F, I**, **5B**). In contrast, for leaves 4–7 at 0–0 or 0–3 dai, there were only small (leaf 4 at 0–0 dai) or either little or no significant differences between these values for virus-mock-inoculated versus systemically virus-infected Surpass 400 plants (**Figures 5A, B**). With Scoop, for 1.05 SBGS, both virus-mock-inoculated and systemically virus-infected leaves always developed 0%LDI values (**Figure 2J**, **5A**). In contrast, at 1.07 SBGS in Scoop, there were either no differences in %LDI values between systemically virus-infected and virus-mock-inoculated leaves or the %LDI values in systemically virus-infected leaves 2 and 3 at 0–0 dai were marginally higher rather than lower as had always occurred in the other two cultivars (**Figures 2K, L**, **5B**). Thus, %LDI outcomes were significantly modulated by leaf maturity, plant age, cultivar fungal resistance, virus infection, and fungal inoculation timing. Older leaves exhibited markedly higher %LDI values than younger leaves, while host resistance (PGR in Scoop and SDGR in Surpass 400) consistently suppressed fungal disease compared with what occurred in cv. Westar (which lacked any fungal resistance). Virus-induced reductions in %LDI values were most prominent in cv. Westar, where mock-inoculated plants sustained the highest disease levels; in Surpass 400, this effect was limited to 0–0 dai. Furthermore, 0–3 dai inoculations yielded higher %LDI values than 0–0 dai, highlighting the critical role of temporal dynamics in pathogen interactions.

### 3.6 Effect of virus infection upon fungal disease severity

**Figure 6:**
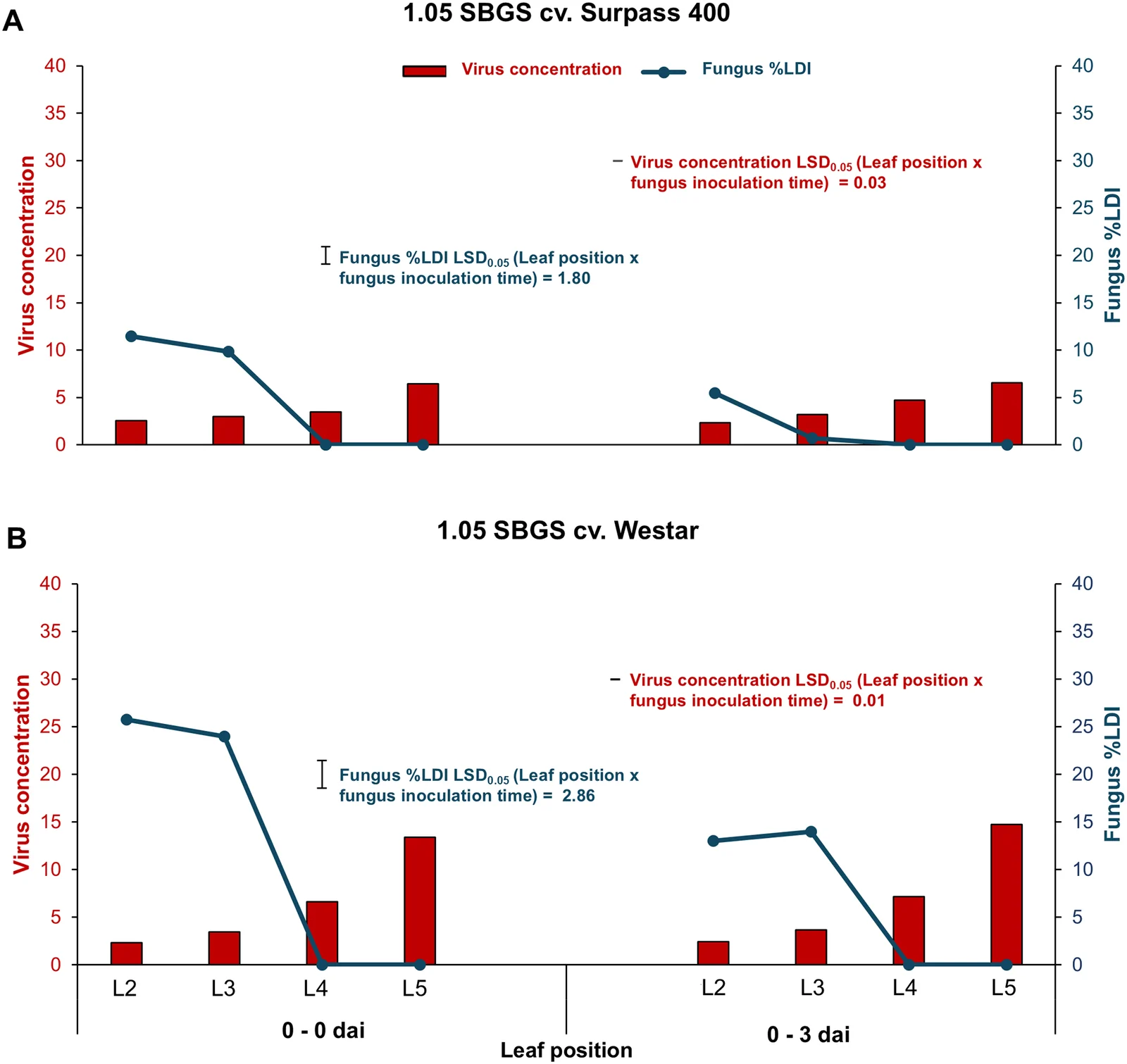
Bar and line chart showing turnip mosaic virus (TuMV) concentration (ELISA optical density exponentially (*e^x^*) transformed A_405_ values) (left axis) and *Leptosphaeria maculans* percentage leaf disease index (%LDI) (right axis) in true leaves that were systemically TuMV-infected or lacking virus infection and present on plants of two canola (*Brassica napus*) cultivars at growth stage 1.05 SBGS. Combined virus concentration (VC) and *L maculans* %LDI values for cotyledon virus-mock-inoculated or TuMV-inoculated cv. Surpass 400 **(A)** or Westar **(B)** plants for each of leaf positions 2–5 at both 0–0 and 0–3 dai, respectively. LSD bars represent significant differences at P < 0.05 between leaf position and *L. maculans* inoculation time. Cultivar resistance/susceptibility status: without *L. maculans* resistance (Westar) and *L. maculans* single dominant gene resistance (SDGR) (Surpass 400). LSD bars represent the significant differences at *P* < 0.05 between leaf position and *L. maculans inoculation* time. To obtain true leaves with or without TuMV infection, cotyledons had previously been inoculated with TuMV-infective sap or virus-mock-inoculated with healthy sap, respectively. The delay between initial cotyledon inoculation with TuMV and the first of the two *L. maculans* true leaf inoculations was 35 days. *L. maculans* drop inoculations were performed on different halves of each true leaf, the first inoculation on day 0 and the second at either of the two *L. maculans* inoculation timings (0–0 and 0–3 dai, respectively). *L. maculans* %LDI values were recorded 11 days after the second *L. maculans* inoculation to canola plants. VC values were from each *L. maculans*-inoculated leaf at positions 2–5 sampled 12 to 13 days after the second *L. maculans* inoculations.

**Figure 7:**
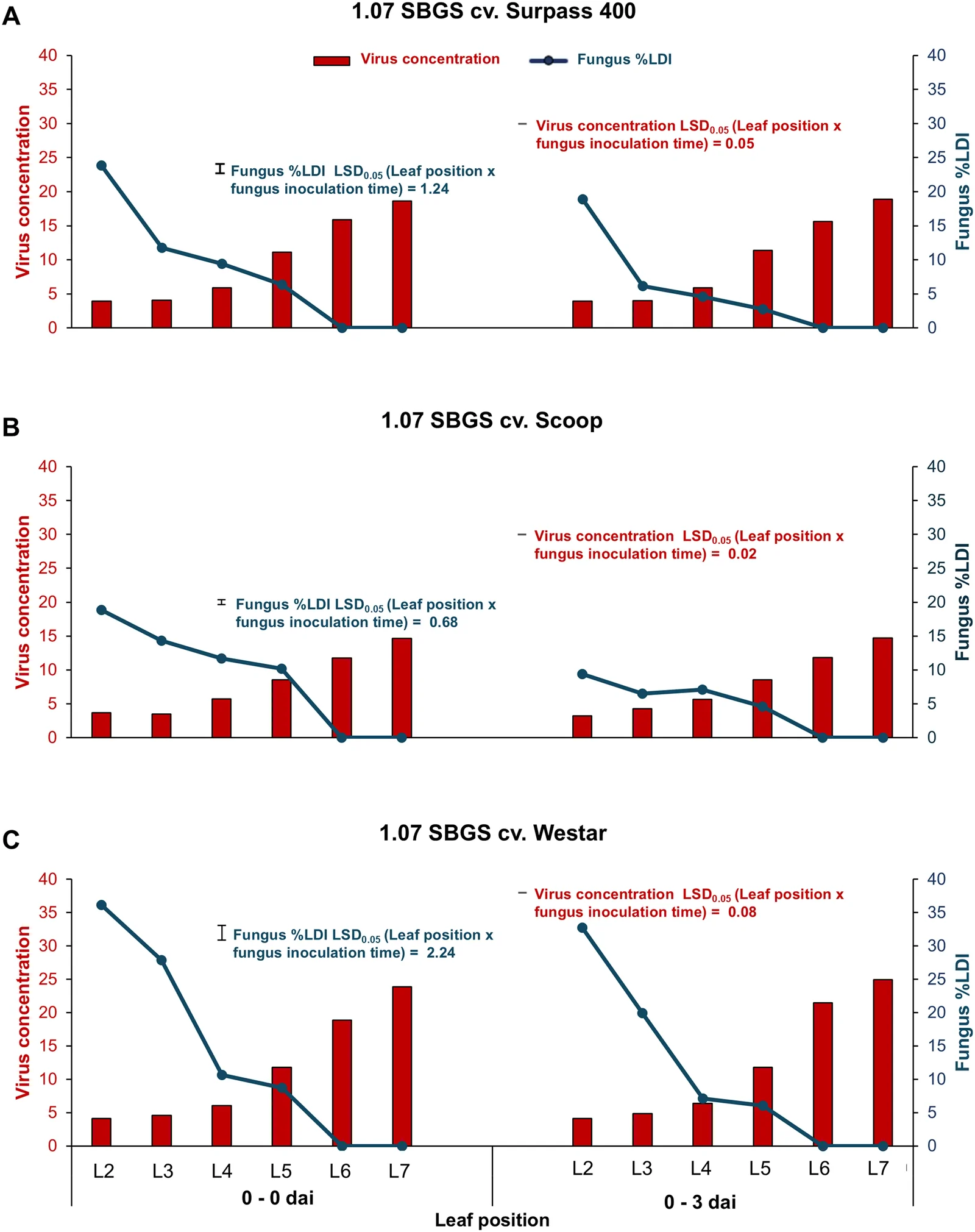
Bar and line chart showing turnip mosaic virus (TuMV) concentration (ELISA optical density exponentially (*e^x^*) transformed A_405_ values) (left axis) and *Leptosphaeria maculans* percentage leaf disease index (%LDI) (right axis) in true leaves systemically TuMV-infected or lacking TuMV infection present on plants of three canola (*Brassica napus*) cultivars at the 1.07 Sylvester–Bradley growth stage (SBGS). Virus concentration (VC) and *L. maculans* %LDI values for cotyledon virus-mock-inoculated or TuMV-inoculated cv. Surpass 400 **(A)**, cv. Scoop **(B)**, and cv. Westar **(C)** plants for each of leaf positions 2–7 at both 0–0 and 0–3 dai, respectively. LSD bars represent the significant differences at *P* < 0.05 between leaf position and *L. maculans* inoculation time. Cultivar resistance/susceptibility status: without *L. maculans* resistance (Westar), *L. maculans* single dominant gene resistance (SDGR) (Surpass 400), and combined *L maculans* polygenic resistance (PGR) and TuMV temperature-sensitive systemic invasion resistance (TSSIR) (Scoop). To obtain the TuMV-infected or healthy leaves, cotyledons had previously been inoculated with TuMV-infective sap or virus-mock-inoculated with healthy sap, respectively. The delay between cotyledon inoculation with TuMV and the first of the two *L. maculans* true leaf inoculations was 50 days. *L. maculans* drop inoculations were performed on different halves of each true leaf, the first inoculation on day 0 and the second at either of the two *L. maculans* inoculation timings (0–0 and 0–3 dai, respectively). *L. maculans* %LDI values were recorded 11 days after the second *L. maculans* inoculations to canola plants. VC values were recorded from true leaves after sampling them 12 to 13 days after the second *L. maculans* inoculation.

Overall, at 1.05 and 1.07 SBGS across all cultivars, inoculation times, and leaf maturity, for true leaves of infected plants, there were significant differences between %LDI and VC values for different cultivars (*P* < 0.001), times of fungal inoculation (*P* < 0.001), and leaf maturity (*P* < 0.001). A comparison of %LDI and VC values revealed opposite trends as leaf position increased up the stem (**Figures 6**, **7**). Thus, %LDI values were highest on lower leaves (2 and 3), but, with one exception, there was 0%LDI on the uppermost two leaves (4 and 5 for 1.05 SBGS and 6 and 7 for 1.07 SBGS), with intermediate values on middle leaves (4 and 5) for 1.07 SBGS. The single exception to this was in Westar virus-mock-inoculated plants at 0–0 and 0–3 dai where leaves inoculated with fungal strain combination RB (first):RB (second) developed symptoms in leaves 4 at 1.05 SBGS and 6 at 1.07 SBGS (**Tables 2A, B**). VC values were least in leaves 2 and 3 and greatest in leaves 4 and 5 for 1.05 SBGS and in leaves 6 and 7, but intermediate in leaves 4 and 5 at 1.07 SBGS (**Figures 6**, **7**).

**Table 2:** Fungal percentage leaf disease index outcomes following canola cotyledon inoculation with turnip mosaic virus (TuMV) followed by true-leaf inoculation with *Leptosphaeria maculans*.

A. Data for the 1.05 Sylvester–Bradley growth stage						
Resistance (cultivar)	Initial virus or virus mock-inoculation	Same day fungus 2nd inoculations (0-0 dai) or fungus mock-inoculation (=Deionized water)	Leaf position			
			L2	L3	L4	L5
SDGR						
(Surpass 400)	Mock	Deionized water	0	0	0	0
		non-RB (1st) - non-RB (2nd)	10.00 ab	5.00	0	0
		non-RB (1st) - RB (2nd)	8.75 ab	7.50	0	0
		RB (1st) - RB (2nd)	46.25 d	37.50	15.00	0
		RB (1st) - non-RB (2nd)	6.88 ab	6.25	0	0
SDGR						
**(Surpass 400)**	**Virus**	**Deionized water**	**0**	**0**	**0**	**0**
		**non-RB (1st) - non-RB (2nd)**	**5.25 a**	**0**	**0**	**0**
		**non-RB (1st) - RB (2nd)**	**4.69 a**	**3.75**	**0**	**0**
		**RB (1st) - RB (2nd)**	**30.00 c**	**31.25**	**0**	**0**
		**RB (1st) - non-RB (2nd)**	**5.00 a**	**4.38**	**0**	**0**
(Westar)	Mock	Deionized water	0	0	0	0
		non-RB (1st) - non-RB (2nd)	67.5 e	46.25	12.50	0
		non-RB (1st) - RB (2nd)	61.25 e	46.25	12.50	0
		RB (1st) - RB (2nd)	61.25 e	43.75	15.00	0
		RB (1st) - non-RB (2nd)	58.75 e	48.75	11.25	0
None						
**(Westar)**	**Virus**	**Deionized water**	**0**	**0**	**0**	**0**
		**non-RB (1st) - non-RB (2nd)**	**20.00 b**	**20.00**	**0**	**0**
		**non-RB (1st) - RB (2nd)**	**31.25 c**	**30.00**	**0**	**0**
		**RB (1st) - RB (2nd)**	**45.00 d**	**40.00**	**0**	**0**
		**RB (1st) - non-RB (2nd)**	**32.50 c**	**30.00**	**0**	**0**

Resistance (cultivar)	Initial virus or virus mock-inoculation	Delayed fungus 2nd inoculations (0-3 dai) or fungus mock-inoculation (=Deionized water)	Leaf position			
			L2	L3	L4	L5
SDGR						
(Surpass 400)	Mock	Deionized water	0	0	0	0
		non-RB (1st) - non-RB (2nd)	7.25 ab	6.50	0	0
		non-RB (1st) - RB (2nd)	5.00 a	5.00	0	0
		RB (1st) - RB (2nd)	30.00 c	31.30	0	0
		RB (1st) - non-RB (2nd)	7.50 ab	5.00	0	0
SDGR						
**(Surpass 400)**	**Virus**	**Deionized water**	**0**	**0**	**0**	**0**
		**non-RB (1st) - non-RB (2nd)**	**5.00 a**	**0**	**0**	**0**
		**non-RB (1st) - RB (2nd)**	**4.38 a**	**3.75**	**0**	**0**
		**RB (1st) - RB (2nd)**	**9.75 ab**	**0**	**0**	**0**
		**RB (1st) - non-RB (2nd)**	**6.25 a**	**0**	**0**	**0**
None						
(Westar)	Mock	Deionized water	0	0	0	0
		non-RB (1st) - non-RB (2nd)	26.25 b	25.00	0	0
		non-RB (1st) - RB (2nd)	55.00 de	35.00	15.00	0
		RB (1st) - RB (2nd)	28.75 c	23.75	0	0
		RB (1st) - non-RB (2nd)	28.75 c	23.75	0	0
None						
**(Westar)**	**Virus**	**Deionized water**	**0**	**0**	**0**	**0**
		**non-RB (1st) - non-RB (2nd)**	**17.50 b**	**17.50**	**0**	**0**
		**non-RB (1st) - RB (2nd)**	**22.50 b**	**20.00**	**0**	**0**
		**RB (1st) - RB (2nd)**	**13.75 ab**	**15.00**	**0**	**0**
		**RB (1st) - non-RB (2nd)**	**21.25 bc**	**17.50**	**0**	**0**
		**LSD_0.05_**	**11.10**	**10.00**	**5.67**	**0**
		**Level of significance**	**< 0.05**	**ns**	**ns**	**ns**

2B. 1.07 Sylvester-Bradley growth stage								
Resistance (cultivar)	Initial virus or virus mock-inoculation	Same day fungus inoculations (0-0 dai) or fungus mock-inoculation (=Deionized water)	Leaf position					
			L2	L3	L4	L5	L6	L7
SDGR								
(Surpass 400)	Mock	Deionized water	0	0	0	0	0	0
		non-RB (1st) - non-RB (2nd)	0 a	0	0	0	0	0
		non-RB (1st) - RB (2nd)	12.50 cd	8.75	7.81	5.00	0	0
		RB (1st) - RB (2nd)	45.00 h	32.50	26.88	13.75	5.63	0
		RB (1st) - non-RB (2nd)	11.88 cd	6.88	4.07	1.25	0	0
SDGR								
**(Surpass 400)**	**Virus**	**Deionized water**	**0**	**0**	**0**	**0**	**0**	**0**
		**non-RB (1st) - non-RB (2nd)**	**15.63 de**	**11.88**	**6.88**	**5.31**	**0**	**0**
		**non-RB (1st) - RB (2nd)**	**18.75 e**	**15.00**	**12.50**	**6.56**	**0**	**0**
		**RB (1st) - RB (2nd)**	**42.50 h**	**23.13**	**20.63**	**13.75**	**0**	**0**
		**RB (1st) - non-RB (2nd)**	**15.00 cde**	**12.50**	**11.25**	**5.31**	**0**	**0**
PGR + TSSIR								
(Scoop)	Mock	Deionized water	0	0	0	0	0	0
		non-RB (1st) - non-RB (2nd)	20.00 e	13.75	10.94	5.94	0	0
		non-RB (1st) - RB (2nd)	18.13 e	12.50	10.31	7.81	0	0
		RB (1st) - RB (2nd)	20.63 ef	15.00	13.13	10.63	0	0
		RB (1st) - non-RB (2nd)	18.75 e	12.50	10.94	9.69	0	0
PGR + TSSIR								
**(Scoop)**	**Virus**	**Deionized water**	**0**	**0**	**0**	**0**	**0**	**0**
		**non-RB (1st) - non-RB (2nd)**	**19.38 e**	**17.19**	**14.06**	**12.81**	**0**	**0**
		**non-RB (1st) - RB (2nd)**	**21.87 ef**	**15.31**	**13.13**	**11.56**	**0**	**0**
		**RB (1st) - RB (2nd)**	**22.50 ef**	**18.44**	**15.00**	**12.81**	**0**	**0**
		**RB (1st) - non-RB (2nd)**	**24.38 f**	**20.52**	**16.19**	**13.75**	**0**	**0**
None								
(Westar)	Mock	Deionized water	0	0	0	0	0	0
		non-RB (1st) - non-RB (2nd)	75.00 m	62.50	18.13	13.13	8.13	0
		non-RB (1st) - RB (2nd)	77.50 mn	64.06	17.19	12.81	8.75	0
		RB (1st) - RB (2nd)	80.00 n	67.19	23.13	14.38	9.69	0
		RB (1st) - non-RB (2nd)	70.00 l	59.38	16.56	14.06	8.75	0
None								
**(Westar)**	**Virus**	**Deionized water**	**0**	**0**	**0**	**0**	**0**	**0**
		**non-RB (1st) - non-RB (2nd)**	**43.75 h**	**31.88**	**13.44**	**10.31**	**0**	**0**
		**non-RB (1st) - RB (2nd)**	**43.13 h**	**33.13**	**12.81**	**10.94**	**0**	**0**
		**RB (1st) - RB (2nd)**	**51.25 i**	**38.13**	**15.31**	**12.81**	**0**	**0**
		**RB (1st) - non-RB (2nd)**	**42.50 h**	**36.25**	**11.56**	**9.38**	**0**	**0**

Resistance (cultivar)	Initial virus or virus mock-inoculation	Delayed fungus 2nd inoculation (0-3 dai) or fungus mock-inoculation (=Deionized water)	Leaf position					
			L2	L3	L4	L5	L6	L7
SDGR								
(Surpass 400)	Mock	Deionized water	0	0	0	0	0	0
		non-RB (1st) - non-RB (2nd)	0 a	0	0	0	0	0
		non-RB (1st) - RB (2nd)	5.63 b	3.75	2.81	1.56	0	0
		RB (1st) - RB (2nd)	39.38 gh	20.00	16.25	10.31	2.19	0
		RB (1st) - non-RB (2nd)	7.81 b	7.81	5.94	4.38	0	0
SDGR								
**(Surpass 400)**	**Virus**	**Deionized water**	**0**	**0**	**0**	**0**	**0**	**0**
		**non-RB (1st) - non-RB (2nd)**	**11.88 c**	**6.25**	**4.69**	**3.13**	**0**	**0**
		**non-RB (1st) - RB (2nd)**	**8.75 bc**	**6.25**	**5.00**	**2.50**	**0**	**0**
		**RB (1st) - RB (2nd)**	**35.00 g**	**10.94**	**8.13**	**4.69**	**0**	**0**
		**RB (1st) - non-RB (2nd)**	**8.13 bc**	**5.63**	**4.38**	**1.88**	**0**	**0**
PGR + TSSIR								
(Scoop)	Mock	Deionized water	0	0	0	0	0	0
		non-RB (1st) - non-RB (2nd)	11.25 cd	9.69	7.81	2.50	0	0
		non-RB (1st) - RB (2nd)	13.13 cd	9.38	8.44	5.00	0	0
		RB (1st) - RB (2nd)	13.75 cd	10.31	8.13	5.94	0	0
		RB (1st) - non-RB (2nd)	11.88 cd	10.94	7.50	5.94	0	0
PGR + TSSIR								
**(Scoop)**	**Virus**	**Deionized water**	**0**	**0**	**0**	**0**	**0**	**0**
		**non-RB (1st) - non-RB (2nd)**	**15.00 de**	**7.50**	**5.94**	**4.06**	**0**	**0**
		**non-RB (1st) - RB (2nd)**	**10.00 c**	**7.81**	**7.19**	**5.94**	**0**	**0**
		**RB (1st) - RB (2nd)**	**11.25 cd**	**9.38**	**7.81**	**7.81**	**0**	**0**
		**RB (1st) - non-RB (2nd)**	**10.63 c**	**7.81**	**6.88**	**5.31**	**0**	**0**
None								
(Westar)	Mock	Deionized water	0	0	0	0	0	0
		non-RB (1st) - non-RB (2nd)	56.25 j	38.13	12.50	10.00	4.38	0
		non-RB (1st) - RB (2nd)	58.75 jk	39.38	11.88	8.44	4.69	0
		RB (1st) - RB (2nd)	61.25 k	43.44	14.38	11.88	5.31	0
		RB (1st) - non-RB (2nd)	50.63 i	32.50	10.63	6.56	5.00	0
None								
**(Westar)**	**Virus**	**Deionized water**	**0**	**0**	**0**	**0**	**0**	**0**
		**non-RB (1st) - non-RB (2nd)**	**37.50 g**	**22.81**	**8.13**	**7.50**	**0**	**0**
		**non-RB (1st) - RB (2nd)**	**77.40 m**	**22.81**	**9.06**	**8.13**	**0**	**0**
		**RB (1st) - RB (2nd)**	**44.38 h**	**29.69**	**10.94**	**8.13**	**0**	**0**
		**RB (1st) - non-RB (2nd)**	**36.25 g**	**24.38**	**7.50**	**6.56**	**0**	**0**
		**LSD_0.05_**	**4.10**	**8.63**	**6.59**	**5.69**	**2.99**	**0**
		**Level of significance**	**< 0.05**	**ns**	**ns**	**ns**	**ns**	**ns**

Initial canola (*Brassica napus*) cotyledon inoculation with TuMV resistance breaking (RB) strain was followed by two subsequent true leaf inoculations with *L. maculans* non-resistance breaking (non-RB) and/or RB strains. The pathogen isolates used were 12.1 for TuMV RB strain, UWA 192 for *L. maculans* RB strain, and P11 for *L. maculans* non-RB strain. The resistance/susceptibility status of the three canola cultivars used was as follows: without any *L. maculans* resistance (Westar), with *L. maculans* single dominant gene resistance (=SDGR) (Surpass 400), and with combined *L. maculans* polygenic resistance (=PGR) and TuMV temperature-sensitive systemic invasion resistance (=TSSIR) (Scoop). The delay between cotyledon inoculation with TuMV and *L. maculans* true leaf inoculation was 35 days at 1.05 SBGS and 50 days at 1.07 SBGS. Second *L. maculans* inoculations were either on the same day as the first (=0–0 dai) or 3 days afterward (=0–3 dai) (i.e., first inoculation at day 0 and second inoculation at day 3). *L. maculans* percentage leaf disease index (%LDI) values were recorded 11 days after the second *L. maculans* inoculation to the three canola cultivars. Different letters indicate significant difference at *P* < 0.05 based on Fisher’s protected LSD. Mock-inoculated means cotyledons inoculated with healthy plant sap. Deionized water was used for fungus mock inoculations.

L2, leaf position 2; L3, leaf position 3; L4, leaf position 4; L5, leaf position 5; L6, leaf position 6; L7, leaf position 7. Bold, virus infected.

At 1.05 SBGS on systemically virus-infected plants, Westar developed very low VC values of 2.4 and 3.5 (0–0 dai) and 2.5 and 3.7 (0–3 dai) for leaves 2 and 3, respectively, compared with leaf 4 (VC of 6.6 at 0–0 dai and 7.2 at 0–3 dai) and leaf 5 (VC of 13.4 at 0–0 and 14.8 at 0–3 dai) (**Figure 6B**). By contrast, leaves 2 and 3 developed high %LDI values of 25.8 and 24.0 (0–0 dai) and 13.0 and 14.0 (0–3 dai), while leaves 4 and 5 had a %LDI value of 0. Similar outcomes and trends were obtained for Surpass 400, but with significantly lower VC and %LDI values for leaves 2 and 3 than those for Westar (**Figure 6A**). At 1.07 SBGS on systemically virus-infected plants, the highest %LDI values occurred on leaf 2 across all cultivars at 0–0 dai. Disease levels subsequently declined through leaf 5, with leaves 6 and 7 consistently exhibiting 0% LDI (**Figures 7A–C**). A comparable trend was observed at 0–3 dai, with the exception of Scoop, which showed equivalent %LDI values between leaves 3 and 4 (**Figure 7B**). For 0–0 dai, the highest %LDI was on Westar, with a declining range of 36.1–8.7 across leaf positions 2–5 and with an ascending VC range from 4.1 to 23.9 across leaf positions 2–7 (**Figure 7C**). This was followed by Surpass 400 with a declining %LDI range of 23.9–6.4 across leaf positions 2–5 and an ascending VC range of 3.9–18.6 across leaf positions 2–10 (**Figure 7A**). Scoop developed the lowest range of declining %LDI values of 18.9–10.2 across leaf positions 2–5 as well as the lowest ascending VC of 3.7–14.7 across leaf positions 2–5 (**Figure 7B**). For Westar, there were significantly lower %LDI and VC values at 0–3 dai compared with those at 0–0 dai, with a %LDI range of 32.8–6.1 across ascending leaf positions 2–5 and VC range of 4.2–23.4 across ascending leaf positions 2–7. With Surpass 400, the corresponding values were %LDI range of 18.9–2.8 across ascending leaf positions 2–5 and VC range of 3.9–18.9 across ascending leaf positions 2–7. With Scoop, they were a %LDI range of 9.4–4.6 across ascending leaf positions 2–5 and VC range of 3.2–14.7 across ascending leaf positions 2–7 (**Figures 7A–C**). Thus, in Westar and Scoop, %LDI values were inversely correlated with VC values, consistently reaching zero in the two youngest leaves (highest VC). SDGR’s presence in Surpass 400 effectively reduced %LDI compared to its values in cv. Westar. In Scoop (PGR and TSSIR), VC values were lower overall but followed a similar decline with leaf maturity, whereas %LDI remained zero at 1.05 SBGS and minimal at 1.07 SBGS, particularly following the 0–3 dai second fungal inoculation.

### 3.7 Effect of fungal strain, virus infection, leaf maturity, and fungal second inoculation time upon fungal disease severity

**Figure 8:**
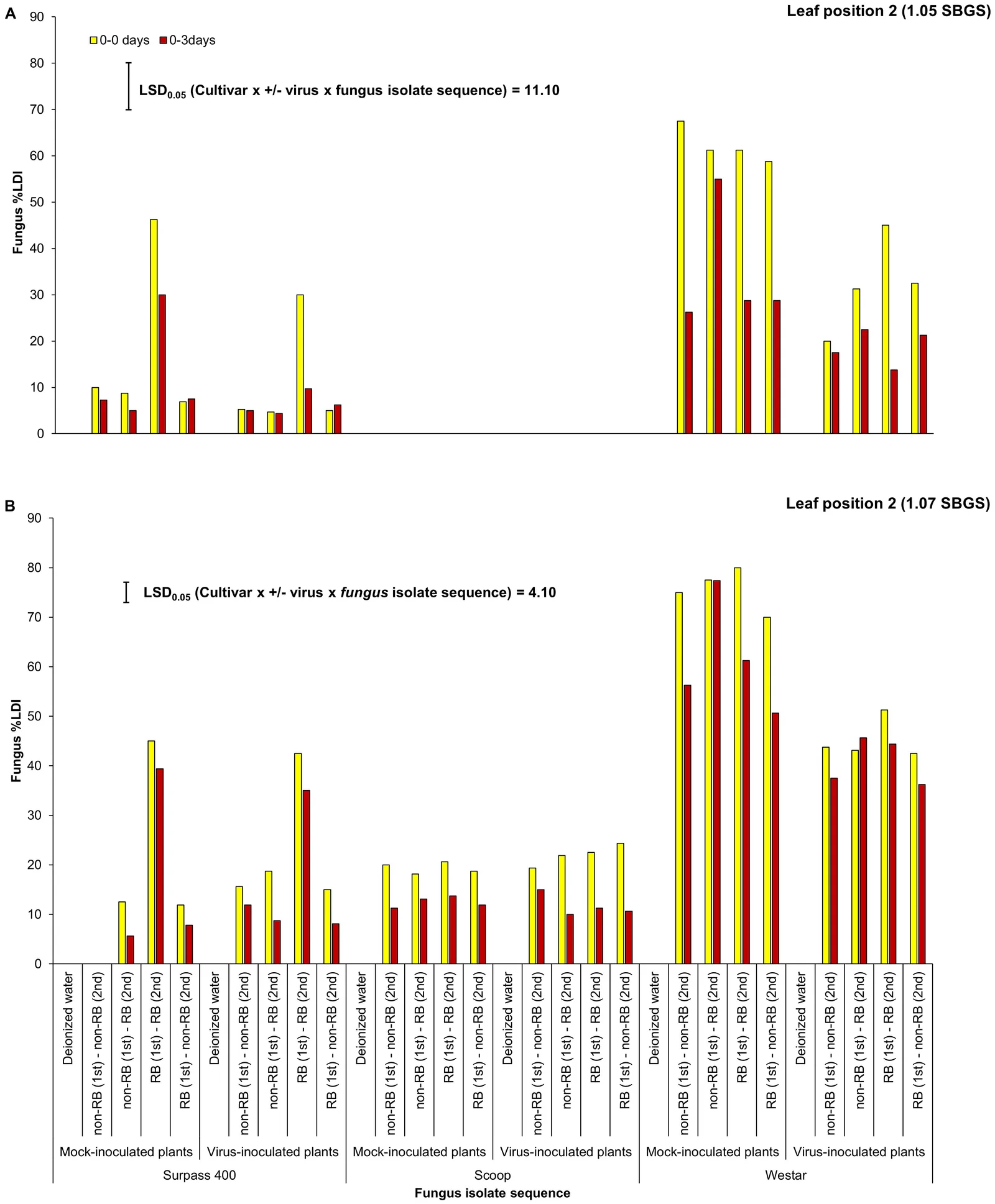
Bar chart showing *Leptosphaeria maculans* percentage leaf disease index (%LDI) values at leaf position 2 in true leaves systemically Turnip mosaic virus (TuMV)-infected or lacking TuMV infection present on plants of three canola (*Brassica napus*) cultivars. **(A)** *L. maculans* %LDI values for leaf position 2 for each individual *L. maculans* strain/isolate inoculation sequence on virus-mock-inoculated or TuMV-infected plants at 1.05 SBGS and second inoculation times (0–0 and 0–3 dai, respectively). **(B)** *L. maculans* %LDI values for leaf position 2 for each individual *L. maculans* strain/isolate inoculation sequence on virus-mock-inoculated or TuMV-infected plants at 1.07 SBGS and second inoculation times (0–0 and 0–3 dai, respectively). LSD bars represent significant differences at *P* <0.05 between cultivar, TuMV presence or absence, and *L. maculans* isolate sequence. Cultivar resistance/susceptibility status: fully fungus susceptible (Westar), *L. maculans* single dominant gene resistance (SDGR) (Surpass 400), and combined *L. maculans* polygenic resistance (PGR) and TuMV temperature-sensitive systemic invasion resistance (TSSIR) (Scoop). *L. maculans* isolates used: non–RB strain isolate P11 and RB strain isolate UWA192. To obtain TuMV-infected or healthy leaves, cotyledons had previously been inoculated with TuMV-infective sap or virus-mock-inoculated with healthy sap, respectively. The delay between initial cotyledon inoculation with TuMV and the first of the two *L. maculans* true leaf inoculations was 35 days at 1.05 Sylvester–Bradley growth stage (SBGS) and 50 days at 1.07 SBGS. *L. maculans* drop inoculations were performed on different halves of each true leaf, the first inoculation on day 0 and the second at either of two *L. maculans* inoculation timings (0–0 and 0–3 dai, respectively). %LDI values were recorded 11 days after *L. maculans* inoculations to canola plants.

The cotyledon virus-mock-inoculated plants were used as a control comparison with the systemically virus-infected plants when both were inoculated with different fungal strain combinations at different true leaf positions and second fungal inoculation times (**Tables 2A, B**). At 1.05 SBGS, only the %LDI values for true leaf position 2 were significantly different (*P* < 0.05) across plants of the different cultivars regardless of whether they were cotyledon virus-mock-inoculated, systemically virus-infected, or inoculated with different fungal strain combinations at either 0–0 or 0–3 dai (**Table 2A**). The same applied at 1.07 SBGS where %LDI values for leaf position 2 were significantly different (*P* < 0.05) across the different cultivars (**Table 2B**). While at both 1.05 and 1.07 SBGS leaf position 2 had the greatest %LDI values compared to other true leaf positions, they were still significantly different (*P* < 0.05) from each other (**Tables 2A, B**) as well as being highly correlated with the total mean value of all true leaf positions (*R*^2^ = 0.99 for 1.05 SBGS; *R*^2^ = 0.98 for 1.07 SBGS). This result for leaf position 2 is highlighted among different individual canola cultivars in **Figures 8A, B**.

Inoculation of Surpass 400 true leaves with fungal strain combination RB (first):RB (second) on the same day (0–0 dai) elicited the highest %LDI values on both virus-mock-inoculated and systemically virus-infected plants at 1.05 and 1.07 SBGS as compared with those involving the non-RB strain (i.e., strain combinations non-RB (first):non-RB (second), non-RB (first):RB (second), and RB (first):non-RB (second). Moreover, the fungal strain combination RB (first):RB (second) %LDI values on virus-mock-inoculated plants were higher than those on systemically virus-infected plants for younger (i.e., at 1.05 SBGS) than older (i.e., at 1.07 SBGS; **Figure 2F**) plants (**Figures 8A, B**). In all instances at 1.07 SBGS, %LDI values on true leaves of both cotyledon virus-mock-inoculated and systemically virus-infected plants were significantly higher after second fungal inoculations at 0–0 dai than 0–3 dai regardless of strain combination (**Figure 7B**). By contrast, for 1.05 SBGS at 0–3 dai, this only occurred with strain combination RB (first):RB (second), with no significant differences being present between %LDI values for fungal second inoculation at 0–0 dai than at 0–3 dai wherever the non-RB strain was present (**Figures 1H**, **7A**). In addition, at 1.05 and 1.07 SBGS, both 0–0 and 0–3 dai inoculations with strain combination RB (first):RB (second) elicited significantly smaller %LDI values on systemically virus-infected than on cotyledon virus-mock-inoculated plants (**Figures 8A, B**). Notably, at 1.07 SBGS in Surpass 400, there was no fungal symptom development with strain combination non-RB (first):non-RB (second) on virus-mock-inoculated plants, but systemically virus-infected plants developed %LDI values of 16% and 12% at 0–0 and 0–3 dai, respectively (**Table 2B**; **Figure 8B**).

No fungal symptoms whatsoever developed on true leaves of Scoop at 1.05 SBGS (**Figure 1J**, **7A**). However, they appeared at 1.07 SBGS (**Figures 2K, L**, **8B**), where, for each fungal strain combination, the %LDI values dropped significantly between same-day second fungal inoculation (0–0 dai) and delayed second inoculation (0–3 dai). For virus cotyledon mock-inoculated plants, they decreased from 20% to 11% (non-RB (first):non-RB (second)), 18% to 13% (non-RB (first):RB (second)), 21% to 14% (RB (first):RB (second)), and 19% to 12% (RB (first):non-RB (second)). For virus-infected plants, they decreased from 19% to 15% (non-RB (first):non-RB (second)), 22% to 10% (non-RB (first):RB (second)), 23% to 11% (RB (first):RB (second)), and 25% to 11% (RB (first):non-RB (second)) (**Figure 8B**).

Fungal inoculation to Westar at 1.05 and 1.07 SBGS revealed that true leaves of systemically virus-infected plants developed lower %LDI values than true leaves of cotyledon virus-mock-inoculated plants, with this effect being greater in younger (i.e., at 1.05 SBGS) than older (i.e., at 1.07 SBGS) plants (**Figures 8A, B**). Analysis of comparisons between true leaf same-day second inoculation (0–0 dai) and delayed second inoculation (0–3 dai) of cotyledon virus-mock-inoculated plants highlighted a significant decline in %LDI values at both 1.05 and 1.07 SBGS (*P* < 0.05) across all strain combinations except non-RB (first):RB (second) (**Figures 8A, B**). On systemically virus-infected plants at 1.05 SBGS, there was a significant decrease in %LDI values resulting from inoculations at 0–0 and 0–3 dai for the fungal strain combinations RB (first):RB (second) (%LDI of 45 to 14) and RB (first):non-RB (second) (%LDI of 33 to 21). However, this decrease was not significant for strain combinations non-RB (first):non-RB (second) (%LDI of 20.0 to 17.5) and non-RB (first):RB (second) (%LDI of 43 and 46) (**Figures 2C**, **8A**). At 1.07 SBGS, there was a significant decrease in %LDI values between true leaf inoculations at 0–0 and 0–3 dai across all of the abovementioned strain/isolate combinations, except when the combination non-RB (first):RB (second) was inoculated to systemically virus-infected plants (**Figure 8B**).

Although not significantly different statistically following second fungal inoculation at 0–3 dai for true leaf 3 at1.05 SBGS and leaf 3 (and, to a lesser extent, leaf 4) at 1.07 SBGS in Surpass 400 and Westar, the same general trends in %LDI values as those obtained in cotyledon virus-mock-inoculated and virus-infected leaf 2 were also evident in them (**Tables 2A, B**). However, their %LDI values were always lower than those in leaf 2. With Surpass 400, strain/isolate combination RB (first):RB (second) elicited the highest %LDI values, RB (first):non-RB (second) and non-RB (first):RB (second) elicited intermediate values, and non-RB (first):non-RB (second) elicited the lowest values. With Westar, strain combination RB (first):RB (second) again elicited the highest %LDI values. In Scoop at 1.07 SBGS, for true leaves of both cotyledon virus-mock-inoculated and systemically virus-infected plants, the %LDI values in leaves 3 to 5 were also lower than those in leaf 2.

In summary, apart from in Scoop (which carries fungal PGR and viral TSSIR) where fungal disease was absent at 1.05 SBGS, %LDI values for true leaf position 2 differed significantly across all three cultivars regardless of being systemically virus-infected or virus-mock-inoculated, fungal strain combination, or second fungus inoculation time. In cv. Westar (which lacks fungal resistances), systemically virus-infected plants generally exhibited lower %LDI values than virus-mock-inoculated plants, revealing virus-induced fungal disease suppression. Furthermore, in virus-mock-inoculated Westar plants at 1.05 and 1.07 SBGS, %LDI values decreased significantly between fungal second inoculations at 0–0 and 0–3 dai with all fungal strain combinations apart from non-RB (first):RB (second). The same trend occurred on systemically virus-infected plants where statistically significant %LDI values decreased significantly between 0–0 and 0–3 dai for most fungal strain combinations, apart from non-RB (first):RB (second) (at 1.05 and 1.07 SBGS) and non-RB (first):non-RB (second) (at 1.05 SBGS). With cv. Surpass 400 (which carries fungal SDGR), on both virus-mock-inoculated or virus-infected plants, inoculation with fungal strain combination RB (first):RB (second) at 1.05 or 1.07 SBGS at 0–0 dai or at 1.07 SBGS at 0–3 dai always elicited higher %LDI values than those obtained with any combination involving non-RB strain. With 1.05 SBGS at 0–3 dai, this occurred with fungal strain combination RB (first):RB (second), but not where non-RB strain was present. Furthermore, at 1.07 SBGS, infection with the combination non-RB (first):non-RB (second) elicited fungal symptoms on systemically virus-infected but not virus-mock-inoculated plants. Therefore, when fungal SDGR was present, virus infection was sometimes able to counteract its ability to suppress %LDI values caused by infection with the non-RB strain. With cv. Scoop, at 1.07 SBGS %LDI values declined between inoculation days 0–0 and 0–3 dai with every fungal strain combination. By contrast, as mentioned above, no fungal symptoms whatsoever developed at 1.05 SBGS. The %LDI values at 1.07 SBGS were similar for each fungal strain combination regardless of whether the plants were virus-mock-inoculated or systemically virus-infected. Therefore, the fungal RB strain was ineffective at counteracting PGR. There was no evidence that the much lower VC values in Scoop than in Westar resulting from TSSIR at 1.07 SBGS (see next section) were enhancing the suppression of %LDI values caused by PGR because the %LDI values in virus-mock-inoculated and virus-infected plants were similar. However, this might possibly have occurred at 1.05 SBGS where the %LDI level was always 0. The five key outcomes arising from this section are listed in **Table 3**.

**Table 3:** Key outcomes from this virus–fungus coinfection research.

For fungal disease following virus infection
(i) In the cultivar lacking fungal disease resistance (Westar), there was marked suppression of fungal disease severity (i.e., an antagonistic response) in coinfected lower leaves compared with leaves infected with the fungus alone (i.e., virus-mock-inoculated) regardless of fungal strain combination. In addition, the severe fungal disease symptoms on the oldest inoculated leaves of systemically virus-infected or virus-mock-inoculated plants contrasted with the increasingly less severe, followed by the complete absence of fungal disease symptoms, as leaf age declined.
(ii) In the cultivar with fungal SDGR (Surpass 400), when compared with the fungal disease severity in the infected plants with the fungus alone, there was marked suppression of fungal disease severity in both coinfected leaves and leaves infected with the fungus alone whenever the fungal strain combination present included its non-resistance breaking strain (non-RB) strain, but much less suppression when its resistance breaking strain (RB) strain was present alone.
(iii) Also, in the cultivar with fungal SDGR, when the fungal RB strain was present alone, there was greater suppression of fungal disease severity in coinfected leaves compared with the disease severity in leaves infected with the fungus alone (especially for 1.05 SBGS at 0–3 dai).
(iv) In the cultivar with both PGR to the fungus and TSSIR to the virus (Scoop), no fungal disease whatsoever developed at 1.05 SBGS, whereas marked suppression of fungal disease severity occurred at 1.07 SBGS (especially at 0–3 dai) regardless of whether the leaves were coinfected with the virus and different combinations of fungal strains or infected with these same strain combinations in leaves infected with the fungus alone.

For virus concentration following fungus infection
(i) In the cultivar lacking fungal disease resistance (Westar), when compared with the VC in leaves infected with the virus alone, increased VC occurred in the leaves of coinfected plants whenever the fungal strain combination inoculated had its RB strain placed first (i.e., a synergistic interaction). Conversely, VC was suppressed whenever the fungal strain combination inoculated had its RB strain placed last (i.e., an antagonistic interaction).
(ii) Also, in the cultivar lacking fungal disease resistance, greater VC suppression developed in the leaves of coinfected plants following second fungal inoculation at 0–3 dai than at 0–0 dai when the different strain combinations inoculated involved first inoculation with the non-RB strain at 1.05 SBGS (i.e., an antagonistic interaction). Conversely, when first inoculation was with the RB strain at 1.07 SBGS, this resulted in greater VC stimulation (i.e., a synergistic response).
(iii) In the cultivar with fungal SDGR (Surpass 400), when compared with the VC in leaves of plants of the fully fungus-susceptible cultivar infected with the virus alone, there was marked suppression of VC whenever the fungal strain combination present included its RB strain, but less suppression of VC in leaves from plants infected with the virus alone or in coinfected plants when only its non-RB strain was present.
(iv) In the cultivar with fungal PGR and viral TSSIR (Scoop), when compared with the VC in leaves of plants of the fully fungus-susceptible cultivar infected with the virus alone, uniformly low VC values developed regardless of leaves being infected with the virus alone or from plants coinfected with different fungal strain combinations.
(v) Also, in the cultivar with PGR and TSSIR, its uniformly low VC values in leaves from plants coinfected with all fungal strain combinations, or infected with the virus alone, were still higher than the lower values in leaves from plants of the cultivar with SDGR when coinfected with any of three fungal strain combinations that included the fungal RB strain at 1.05 SBGS. Conversely, at 1.07 SBGS, all of these four VC values for the cultivar with PGR and TSSIR were lower than the values these three fungal strain combinations that included the fungal RB strain for the cultivar with SDGR.

### 3.8 Effect of fungal strain, leaf maturity, and second inoculation time upon virus concentration

**Figure 9:**
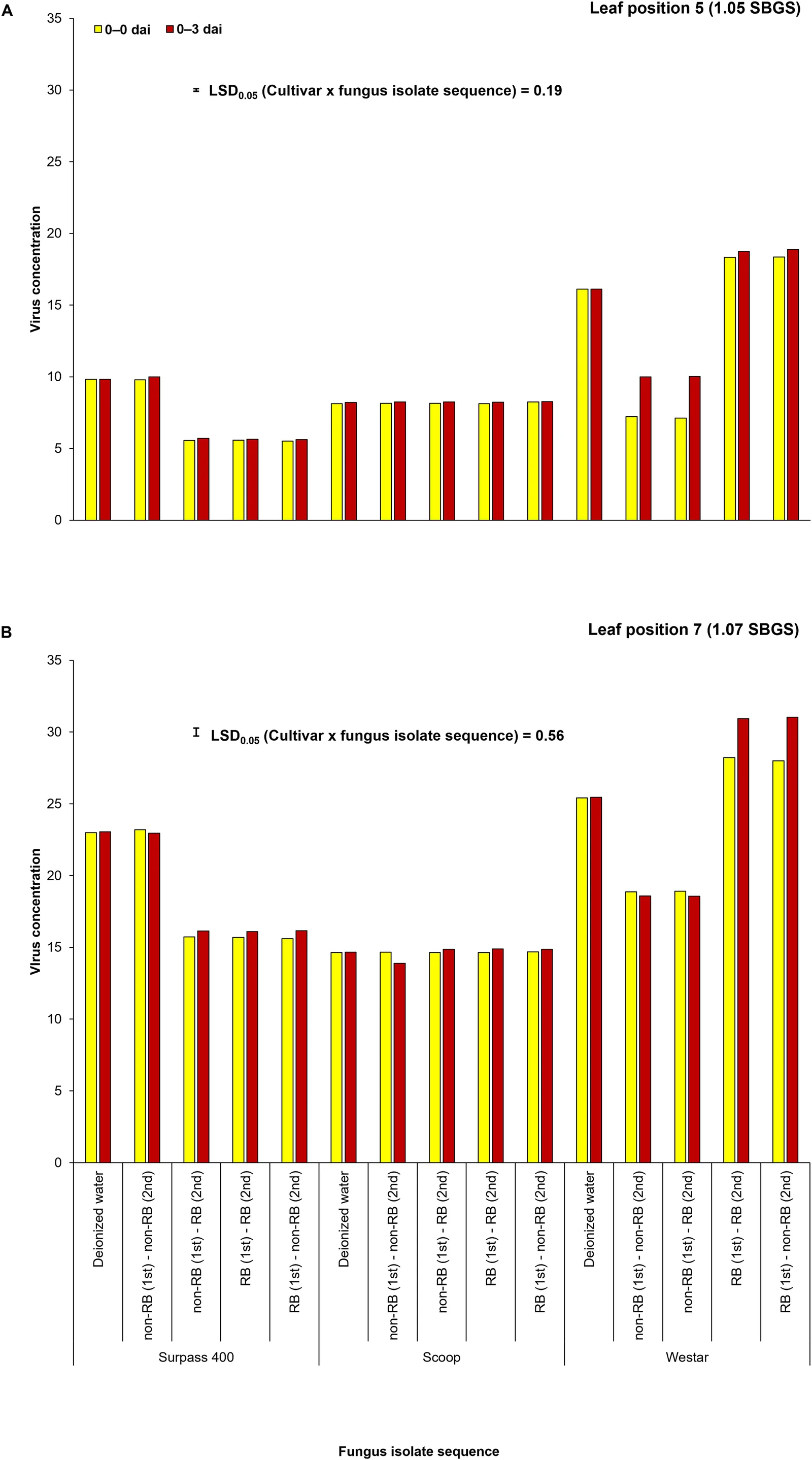
Bar chart showing virus concentration (VC) ELISA optical density exponentially (*e^x^*) transformed A_405_ values at true leaf positions 5 and 7 on systemically turnip mosaic virus (TuMV)-infected or cotyledon mock-inoculated plants of three canola (Brassica napus) cultivars at 1.05 and 1.07 Sylvester–Bradley growth stages (SBGS), respectively. **(A)** VC values for each individual *L. maculans* strain/isolate inoculation sequence on virus-mock-inoculated or TuMV-infected plants at 1.05 SBGS and inoculation times (0–0 and 0–3 dai, respectively). **(B)** VC values for each individual *L. maculans* strain/isolate second inoculation sequence on virus-mock-inoculated or TuMV-infected plants at 1.07 SBGS and second inoculation times (0–0 and 0–3 dai, respectively). LSD bars represent significant differences at *P* <0.05 between cultivar, TuMV presence or absence, and *L. maculans* strain/isolate sequence. Cultivar resistance/susceptibility status: fully fungus susceptible (Westar), *L. maculans* single dominant gene resistance (SDGR) (Surpass 400), and combined *L. maculans* polygenic resistance (PGR) and TuMV temperature-sensitive systemic invasion resistance (TSSIR) (Scoop). *L. maculans* isolates used in the study: non-RB isolate P11 and RB isolate UWA192. To obtain these TuMV-infected or healthy true leaves, cotyledons had previously been inoculated with TuMV-infective sap or virus-mock-inoculated with healthy sap, respectively. The delay between initial cotyledon inoculation with TuMV and the first of the two *L. maculans* true leaf inoculations was 35 days at 1.05 SBGS and 50 days at 1.07 SBGS. *L. maculans* drop inoculations were performed on different halves of each true leaf, the first inoculation on day 0 and the second at either of two *L. maculans* inoculation timings (0–0 and 0–3 dai, respectively). VC values were from inoculated leaf from position 5 (at 1.05 SBGS) and position 7 (at 1.07 SBGS) at 12 to 13 days after the second *L. maculans* inoculations.

Plants systemically virus-infected but without any fungal infection were used as a control to determine how the different fungal strain combinations influenced VC values (**Table 2A**). There was a significant (*P* < 0.001) four-way interaction across cultivars, fungal strain combinations, leaf positions, and second fungal inoculation times 0–0 or 0–3 dai in relation to the resulting VC values at 1.05 and 1.07 SBGS (**Tables 4A, B**). The VC values recorded for each true leaf position were significantly different across cultivars, fungal strain combinations, and second fungal inoculation times at 0–0 or 0–3 dai. The only exceptions were leaf position 4 for 1.05 SBGS (*P* > 0.05; **Tables 4A, B**) and leaf position 2 for 1.07 SBGS (*P* > 0.05; **Table 4B**). Leaf position 5 for 1.05 SBGS and leaf position 7 for 1.07 SBGS developed the highest VC values compared with all other leaf positions (*P* < 0.001) and were highly correlated with the mean values for all leaf positions (*R*^2^ = 0.93 for 1.05 SBGS and *R*^2^ = 0.95 for 1.07 SBGS). What occurred with these two leaf positions (i.e., leaf position 5 for 1.05 and leaf position 7 for 1.07 SBGS) is highlighted in **Figures 9A, B**. There was a significant difference between the VC values for leaf position 5 at 1.05 SBGS (*P* < 0.001) and leaf position 7 at 1.07 SBGS (*P* < 0.001) compared with other true leaf positions. Nevertheless, similar patterns developed within each cultivar at 1.05 and 1.07 SBGS, but those at 1.07 SBGS developed higher VC values overall than those at 1.05 SBGS (**Figures 9A, B**; **Tables 4A, B**).

**Table 4:** Virus concentration outcomes following cotyledon inoculation with turnip mosaic virus (TuMV) followed by true leaf inoculation with *Leptosphaeria maculans*.

A. Data for virus concentration at 1.05 SBGS						
Resistance (cultivar)	Same day 2nd fungus inoculation (0–0 dai)	Leaf position				Mean
		L2	L3	L4	L5	
SDGR						
(Surpass 400)	Deionized water (virus only)	3.39	3.99	4.47	9.82	5.42 f
	Non-RB (first)–non-RB (second)	3.36	4.02	4.49	9.79	5.42 f
	Non-RB (first)–RB (second)	2.01	2.27	2.78	5.56	3.16 a
	RB (first)–RB (second)	2.02	2.26	2.75	5.57	3.15 a
	RB (first)–non-RB (second)	1.99	2.26	2.77	5.52	3.14 a
PGR + TSSIR						
(Scoop)	Deionized water (virus only)	3.58	3.32	5.69	8.12	5.18 e
	Non-RB (first)–non-RB (second)	3.59	3.39	5.75	8.14	5.22 e
	Non-RB (first)–RB (second)	3.53	3.35	5.73	8.14	5.19 e
	RB (first)–RB (second)	3.55	3.41	5.75	8.13	5.21 e
	RB (first)–non-RB (second)	3.56	3.40	5.69	8.25	5.23 e
None						
(Westar)	Deionized water (virus only)	2.89	4.12	8.50	16.11	7.91 g
	Non-RB (first)–non-RB (second)	1.41	2.28	3.65	7.22	3.64 c
	Non-RB (first)–RB (second)	1.49	2.30	3.69	7.11	3.65 c
	RB (first)–RB (second)	2.99	4.32	8.69	18.33	8.58 h
	RB (first)–non-RB (second)	3.01	4.30	8.71	18.34	8.59 h

Resistance (cultivar)	Delayed 2nd fungus inoculation (0–3 dai)	Leaf position				Mean
		L2	L3	L4	L5	
SDGR						
(Surpass 400)	Deionized water (virus only)	3.40	4.01	4.47	9.83	5.43 f
	Non-RB (first)–non-RB (second)	3.48	4.12	4.72	10.00	5.58 f
	Non-RB (first)–RB (second)	1.79	2.32	3.91	5.69	3.43 b
	RB (first)–RB (second)	1.80	2.29	3.95	5.63	3.42 b
	RB (first)–non-RB (second)	1.74	2.29	3.97	5.62	3.41 b
PGR + TSSIR						
(Scoop)	Deionized water (virus only)	3.50	3.36	5.71	8.20	5.19 e
	Non-RB (first)–non-RB (second)	3.49	3.36	5.75	8.24	5.21 e
	Non-RB (first)–RB (second)	3.43	3.36	5.73	8.25	5.19 e
	RB (first)–RB (second)	3.40	3.41	5.73	8.23	5.19 e
	RB (first)–non-RB (second)	3.42	3.42	5.69	8.27	5.20 e
None						
(Westar)	Deionized water (virus only)	2.90	4.11	8.53	16.10	7.91 g
	Non-RB (first)–non-RB (second)	1.31	2.38	4.55	9.99	4.56 d
	Non-RB (first)–RB (second)	1.33	2.40	4.66	10.01	4.60 d
	RB (first)–RB (second)	3.39	4.72	9.09	18.73	8.98 i
	RB (first)–non-RB (second)	3.41	4.75	9.11	18.88	9.04 i
	LSD_0.05_	0.09	0.12	0.26	0.19	0.17
	Level of significance	<0.001	<0.001	ns	<0.001	<0.001

B: Virus concentration at 1.07 SBGS								
Resistance (cultivar)	Same day fungus inoculation (0–0 dai)	Leaf position						Mean
		L2	L3	L4	L5	L6	L7	
SDGR								
(Surpass 400)	Deionized water (virus only)	4.88	5.18	6.17	13.58	21.00	23.00	12.30 e
	Non-RB (first)–non-RB (second)	4.89	5.16	6.19	13.68	21.05	23.19	12.36 e
	Non-RB (first)–RB (second)	3.33	3.35	5.74	9.40	12.48	15.74	8.34 bc
	RB (first)–RB (second)	3.14	3.25	5.70	9.44	12.44	15.69	8.28 b
	RB (first)–non-RB (second)	3.29	3.31	5.68	9.42	12.41	15.60	8.29 b
PGR + TSSIR								
(Scoop)	Deionized water (virus only)	3.68	3.53	5.72	8.52	11.77	14.64	7.98 b
	Non-RB (first)–non-RB (second)	3.66	3.39	5.71	8.55	11.74	14.66	7.95 b
	Non-RB (first)–RB (second)	3.68	3.55	5.73	8.54	11.75	14.65	7.98 b
	RB (first)–RB (second)	3.65	3.44	5.78	8.53	11.75	14.64	7.97 b
	RB (first)–non-RB (second)	3.66	3.43	5.73	8.55	11.78	14.68	7.97 b
None								
(Westar)	Deionized water (virus only)	5.05	5.71	7.73	14.40	20.64	25.42	13.16 f
	Non-RB (first)–non-RB (second)	2.49	2.60	4.20	8.23	11.79	18.87	8.03 b
	Non-RB (first)–RB (second)	2.51	2.65	4.15	8.20	11.74	18.92	8.03 b
	RB (first)–RB (second)	5.20	6.02	7.02	13.99	25.17	28.22	14.27 g
	RB (first)–non-RB (second)	5.21	6.01	6.99	14.02	25.14	27.99	14.23 g

Resistance (cultivar)	Delayed 2nd fungus inoculation (0–3 dai)	Leaf position						Mean
		L2	L3	L4	L5	L6	L7	
SDGR								
(Surpass 400)	Deionized water (virus only)	4.81	5.16	6.16	13.60	21.02	23.05	12.30 e
	Non-RB (first)–non-RB (second)	4.83	5.16	6.19	13.69	21.06	22.96	12.32 e
	Non-RB (first)–RB (second)	3.28	3.26	5.74	9.87	11.99	16.14	8.38 bcd
	RB (first)–RB (second)	3.28	3.23	5.69	9.86	11.93	16.10	8.35 bcd
	RB (first)–non-RB (second)	3.31	3.20	5.73	9.89	11.98	16.17	8.38 bcd
PGR + TSSIR								
(Scoop)	Deionized water (virus only)	3.69	3.55	5.75	8.56	11.79	14.66	8.00 b
	Non-RB (first)–non-RB (second)	2.66	3.52	4.39	7.02	11.19	13.88	7.11 a
	Non-RB (first)–RB (second)	3.33	4.62	5.93	8.94	11.96	14.87	8.28 b
	RB (first)–RB (second)	3.35	4.65	5.98	8.99	11.95	14.89	8.30 b
	RB (first)–non-RB (second)	3.35	4.65	5.93	8.99	11.98	14.88	8.30 b
None								
(Westar)	Deionized water (virus only)	5.06	5.73	7.75	14.45	20.66	25.46	13.19 f
	Non-RB (first)–non-RB (second)	2.61	3.15	4.90	8.15	15.19	18.58	8.76 c
	Non-RB (first)–RB (second)	2.63	3.17	5.03	8.19	15.15	18.56	8.79 cd
	RB (first)–RB (second)	5.20	6.02	7.02	13.99	28.17	30.93	15.22 h
	RB (first)–non-RB (second)	5.19	6.00	7.04	14.04	28.22	31.03	15.25 h
	LSD_0.05_	0.70	0.32	0.31	0.28	0.46	0.56	0.44
	Level of significance	ns	<0.05	<0.001	<0.001	<0.001	<0.001	<0.001

Initial canola (*Brassica napus*) cotyledon inoculation with TuMV resistance breaking (RB) strain was followed by two subsequent true leaf inoculations with *L. maculans* non-resistance breaking (non-RB) and/or RB strains. The pathogen isolates used were 12.1 for TuMV RB strain, UWA192 for *L. macu*lans RB strain, and P11 for *L. maculans* non-RB strain. The resistance/susceptibility status of the three canola cultivars used was as follows: without any *L. maculans* resistance (Westar), with *L. maculans* single dominant gene resistance (=SDGR) (Surpass 400), and with combined *L. maculans* polygenic resistance (=PGR) and TuMV temperature-sensitive systemic invasion resistance (=TSSIR) (Scoop). The delay between cotyledon inoculation with TuMV and *L. maculans* true leaf inoculation was 35 days at 1.05 SBGS and 50 days at 1.07 SBGS. Second *L. maculans* inoculations were either on the same day as the first (=0–0 dai) or 3 days afterward (=0–3 dai) (i.e., first inoculation at day 0 and second inoculation at day 3). ELISA optical density exponentially (*e^x^*) transformed A_405_ values were recorded 11 days after the second *L. maculans* inoculation to the three canola cultivars. Different letters indicate significant difference *P* < 0.05 based on Fisher’s protected LSD. Deionized water was used for fungus mock inoculations.

L2, leaf position 2; L3, leaf position 3; L4, leaf position 4; L5, leaf position 5; L6, leaf position 6; L7, leaf position 7.

The VC values obtained with Surpass 400 true leaf 5 at 1.05 SBGS revealed no significant differences between systemically virus-infected plants, fungus-mock-inoculated plants at 0–0 or 0–3 dai (9.8 and 9.8), and plants inoculated with fungal strain combination non-RB (first):non-RB (second) (9.8 and 10.0) (**Tables 4A, B**; **Figures 9A, B**). By contrast, following fungal inoculations at both 0–0 or 0–3 dai, the VC values obtained with strain combination sequences non-RB (first):RB (second) (5.6 and 5.7), RB (first):RB (second) (5.6 and 5.6), and RB (first):non-RB (second) (5.5 and 5.6) were all significantly lower (*P* < 0.001) than those in plants with fungus mock inoculation (**Figure 9A**). A similar pattern developed with true leaf 7 at 1.07 SBGS at both 0–0 or 0–3 dai, with no significant differences in VC values occurring between those for fungus mock inoculation (23.0 and 23.1) and those inoculated with fungal strain combination non-RB (first):non-RB (second) (23.2 and 23.0). However, their VC values were significantly higher than those that developed with fungal strain combinations non-RB (first):RB (second) (15.7 and 16.1), RB (first):RB (second) (15.7 and 16.1), and RB (first):non-RB (second) (15.6 and 16.2) (**Table 4B**; **Figure 9B**).

Westar behaved differently from Surpass 400 and Scoop in that its VC values in leaves 5 (at 1.0 SBGS) and 7 (at 1.07 SBGS) were significantly reduced when systemically virus-infected plants of this cultivar were inoculated with the non-RB fungal strain first [combinations non-RB (first):non-RB (second) or non-RB (first):RB (second)] compared with its VC values in leaves 5 or 7 for fungus mock inoculation. However, they were significantly higher whenever the RB strain was inoculated first (combinations RB (first):non-RB (second) or RB (first):RB (second)) (**Tables 4A, B**; **Figures 8A, B**). Moreover, differences arose for both 1.05 and 1.07 SBGS in Westar between different inoculation timings. The leaf 5 0–3 dai inoculations involving non-RB strain alone or first at 1.05 SBGS elicited significantly higher VC values compared with the 0–0 dai inoculations (non-RB (first): non-RB (second), 10.0 versus 7.2, respectively, and non-RB (first):RB (second), 10.0 versus 7.1), respectively. In addition, the leaf 7 0–3 dai inoculations involving RB strain alone or RB strain first at 1.07 SBGS elicited significantly higher VC values compared with the 0–0 dai inoculations (RB (first):RB (second), 30.9 versus 28.2, respectively, and RB (first):non-RB (second) at 31.0 versus 28.0, respectively).

Since Scoop always developed significantly lower VC values at both 0–0 and 0–3 dai with fungus-mock-inoculated leaves than with Surpass 400 and, especially, Westar (**Figures 8A, B**), its VC values demonstrated suppression of virus multiplication by TSSIR. Its true leaf VC values ranged from 8.1 to 8.2 for 1.05 SBGS and 13.9-14.9 for 1.07 SBGS and were the lowest VC values obtained compared with those for Surpass 400 and Westar, except with the three fungal strain combinations involving the RB strain (RB (first):RB (second), RB (first):non-RB (second), and non-RB (first):RB (second)) for 1.05 SBGS at both 0–0 and 0–3 dai for Surpass 400 and the two strain combinations in which the fungal non-RB strain was inoculated first (non-RB (first):non-RB (second) and non-RB (first):RB (second)) with 1.07 SBGS at 0–0 dai for Westar (**Figures 9A, B**). This reflected the reverse scenario for leaf 2 %LDI values in systemically virus-infected plants of Surpass 400 for 1.05 SBGS at 0–0 and 0–3 dai which were lower in all combinations involving the non-RB strain (non-RB (first):non-RB (second), non-RB (first):RB (second), and RB (first):non-RB (second)) (**Figures 8A, B**).

At both 0–0 and 0–3 dai, with true leaves 2–5 at 1.05 SBGS and 2–7 at 1.07 SBGS, the same trends in VC values were evident in both Surpass 400 and Westar (**Tables 4A, B**). With Surpass 400, fungus mock inoculation and inoculation with fungal strain combination non-RB (first):non-RB (second) consistently elicited the highest VC values, whereas the three strain combinations that included the RB strain all elicited lower VC values which were similar to each other. With Westar, fungus mock inoculation and both strain combinations that included the RB strain first elicited the highest VC values, whereas the two combinations involving the non-RB strain first elicited the lowest VC values consistently. Therefore, these same trends that occurred regardless of whether mixed infection with the fungus was present in lower leaves or upper leaves were being remotely influenced by the fungus present in mixed infection in lower leaves, as in leaf 5 at 1.05 SBGS and leaves 6 and 7 at 1.07 SBGS. In Scoop, the VC values were always similar in each leaf position regardless of whether the fungus was inoculated at 0–0 or 0–3 dai or fungus mock inoculation was used.

In summary, within each of the three cultivars, the same trends in VC values were evident regardless of whether the systemically virus-infected leaves were being influenced directly via a mixed infection with the fungus causing necrotic symptoms or remotely from fungus-infected lower leaves. In cv. Westar (which lacks fungal resistance), VC values were always suppressed when the fungal non-RB strain was inoculated first but were higher when fungus mock inoculation was used or when the fungal RB strain was the first to be inoculated. By contrast, the SDGR which cv. Surpass 400 carries suppressed VC values whenever the fungal RB strain was present but not when its non-RB strain was present alone (i.e., non-RB (first):non-RB (second)), which resulted in VC values resembling those resulting from fungus mock inoculation. The contrasting results with these two cultivars demonstrate that, in the absence of SDGR (i.e., in Westar), VC values were suppressed by first inoculation with the fungal non-RB strain but increased by first inoculation with the less virulent RB strain, whereas in SDGR’s presence (i.e., in Surpass 400) VC was suppressed whenever the RB strain was present but not when the non-RB strain was present alone. In Scoop (which carries fungal PGR and viral TSSIR), VC values were lower overall compared with those in Westar and Surpass 400 at 1.07 SBGS reflecting TSSIR presence. However, at 1.05 SBGS, whenever the fungal non-RB strain was present in Surpass 400, the resulting VC values were lower than those in Scoop. Conversely, in systemically virus-infected leaves of Surpass 400 fungal disease (i.e., %LDI values) was always lower whenever the non-RB strain was present. Moreover, the suppressed VC values that Scoop developed were due to TSSIR, but not PGR at 1.05 SBGS because fungal infection was absent and at 1.07 SBGS because they were similar regardless of whether fungal infection was absent or present. The five key outcomes arising from this section are listed in **Table 3**.

## 4 Discussion

This research contributes important new knowledge concerning the risks of relying upon single-pathogen host resistance. We show that virus–fungus interactions can significantly alter pathogen resistance effectiveness. These effects vary depending upon whether the pathogen strains are resistance breaking (RB) or non-resistance breaking (non-RB), the specific resistance profile of the crop cultivar, the maturity and position of the infected leaves, and the age of the infected plant. Using the three canola pathogen strains and cultivars employed previously by Abidin et al. (2025) as a model system, we found that, when single pathogen (the virus)-infected plants or healthy plants of a fully fungus-susceptible cultivar were inoculated with a second pathogen (the fungus), the presence of the first pathogen greatly suppressed the disease caused by the second pathogen regardless of its strain combination, but with the extent of suppression being greater in younger (at 1.05 SBGS) than older (at 1.07 SBGS) plants. For the cultivar with fungal SDGR, when first (virus) pathogen-infected or healthy plants were inoculated with the second (fungus) pathogen, the presence of the first pathogen suppressed the disease caused by the second. Moreover, this suppression was greatest when the second pathogen’s non-RB strain was present. When the second pathogen’s RB strain was present, the extent of suppression was much less, being most evident in younger plants. Such effects were absent in the cultivar with both PGR and TSSIR in which second pathogen disease symptom suppression was similar regardless of first pathogen presence or absence. In addition, introduction of the second pathogen altered the amount of the first pathogen present. In the cultivar lacking any fungus resistance, subsequent infection with second pathogen strain combinations in which its non-RB or RB strains were inoculated first, decreased (i.e., an antagonistic interaction) or increased (i.e., a synergistic interaction) the amount of first pathogen present, respectively. In the cultivar with SDGR, in plants infected with the first pathogen, subsequent inoculation with second pathogen strain combinations that included its RB strain decreased the amount of the first pathogen present, whereas inoculation solely with its non-RB strain failed to alter the amount of the first pathogen. Such effects were absent in the cultivar with both PGR and TSSIR in which second pathogen presence or absence failed to alter the extent of first pathogen suppression.

When a systemically virus-infected plant subsequently becomes infected with a fungal pathogen, the most common outcome is that the severity of the fungal disease symptoms is magnified. However, in a few instances, the opposite outcome occurs when fungal disease severity is reduced or host resistance against it is increased (see Abidin et al. (2025) and references therein). Our coinfection study in which a fungal pathogen was inoculated to leaves of plants of a cultivar lacking fungal resistance that was already systemically virus-infected provides another example of the latter. Factors likely to drive such an occurrence include the combination of pathogen strains present, host genetics, leaf and plant maturity, environmental factors, and interactions between the two pathogens and the host (Seabloom et al., 2010; Tollenaere et al., 2016). While virus infection can prime the host immune response to resist fungal colonization, young, actively growing leaves may be inherently less vulnerable than mature tissues (Hu and Yang, 2019). Moreover, certain nutritional and environmental conditions can strengthen host defense systems against secondary infections. Immune enhancement is likely to be activated by interactions between non-RB pathogen strains and host resistance genes. Such interactions can trigger salicylic acid (SA) and jasmonic acid (JA) pathways (Robert-Seilaniantz et al., 2011; Thaler et al., 2012) in stimulating pathogenesis-related (PR) protein production, followed by systemic acquired resistance (SAR) and cell wall reinforcement (Ding et al., 2022; Kaur et al., 2022; Shobade et al., 2025). Within *Brassica* pathosystems, TuMV infection induces marked changes in host transcriptomics and proteomics, redirecting resources toward broad plant–pathogen interaction pathways (Lyu et al., 2020) and actively modulating PR protein cascades, such as apoplastic PR-1 and plasmodesmata-localized PR-5 proteins (Whitham et al., 2003; He et al., 2025). Within the TuMV–canola pathosystem, these responses are heavily guided by interactions between viral symptom determinants and host genetics (Jenner et al., 2002, Jenner et al., 2003) deploying downstream SA pathways regulated by the canonical *BnaNPR1* architecture (Wang et al., 2020). SAR then spreads systemically from coinfected leaves to protect remote leaves only infected by a single pathogen or operate within parts of coinfected plant tissues only infected by a single pathogen.

To explain how viral pre-infection reduces subsequent fungal damage, we propose an antagonistic priming model. Within this model, initial TuMV colonization establishes a high-baseline SA state which fungal effectors are unable to override, successfully blocking the fungal pathogen during its early, SA-sensitive biotrophic phase (Takahashi et al., 2004; Nováková et al., 2016; Yang et al., 2021). Alternatively, a synergistic co-activation model could suggest that a dual-pathogen challenge may accelerate a multi-tiered, balanced immune deployment rather than enforcing a strict trade-off, a phenomenon supported by recent evidence showing that resistant canola genotypes can simultaneously upregulate both SA and JA branches during early infection (Becker et al., 2017; Hou and Tsuda, 2022). Additionally, although plant cell wall reinforcement via plasmodesmatal callose deposition is a well-established defense mechanism to limit cell-to-cell viral spread (Carrasco-López et al., 2025), in canola, coinfection with the resistance breaking strain of TuMV did not prevent penetration by fungal hyphae (Abidin et al., 2025). Insights from *Arabidopsis* suggest that TuMV actively subverts this barrier, the virus selectively upregulating host cell wall protein AtOSM34 which suppresses callose plasmodesmata deposition and thus enabling unhindered viral movement into adjacent uninfected cells (He et al., 2025).

A future study is warranted to provide experimental evidence, such as qPCR and northern blot transcript analysis of marker genes like PR1 or PDF1.2 (Spoel et al., 2003; Pečenková et al., 2017; Pieterse et al., 2012; Van der Does et al., 2013) to confirm that the SA and JA pathways are being triggered in this way by coinfection of canola with the viral and fungal pathogens we studied here, thereby validating either the antagonistic or synergistic models. In addition, higher concentrations of glucose and fructose develop in younger leaves, but greater sucrose and starch reserves accumulate in older leaves (Picanço et al., 2023). Because soluble sugar levels regulate immune activation, this distinct carbohydrate partitioning likely influences the altered fungal severity and infection failures observed between young and mature leaves (Morkunas and Ratajczak, 2014; Trouvelot et al., 2014). Soluble sugar levels regulate immune activation, which can stimulate both infection failure and changes in fungal disease severity differences between young and mature leaves (Morkunas and Ratajczak, 2014; Trouvelot et al., 2014).

Here we found that the fully fungus-susceptible cultivar developed systemic virus symptoms fastest, the cultivar with fungal SDGR next, and the cultivar with both fungal PGR and viral TSSIR last, establishing a linkage between disease symptom progression and host genetics (Kumar et al., 2025). Furthermore, after fungal inoculation, the fully fungus-susceptible cultivar developed the most severe fungal disease symptoms, followed by the cultivar with SDGR, with the least severe fungal disease symptoms developing in the cultivar with both fungal PGR and viral TSSIR. Fungal disease severity depended upon leaf position, being greatest on the oldest co-inoculated true leaf (leaf 2) but disappearing completely on the youngest true leaves (leaf 5 in younger plants and leaf 7 in older plants). By contrast, the differences in VC values were repeated across cultivars regardless of true leaf position despite being highest in youngest upper leaves and lowest in oldest lower leaves. In addition, they occurred regardless of whether these leaves were coinfected or influenced remotely from the fungal infection in coinfected older leaves. Subsequent fungal inoculations onto systemically virus-infected leaves confirmed that host resistance, pathogen strain, tissue maturity, plant age, cultivar type, and the timing of the secondary inoculation challenge all dictate both fungal symptom development and final viral concentration. Crucially, the decline in fungal disease severity matched an increasing VC gradient. This pattern reflects the systemic movement of the virus through the phloem from inoculated cotyledons, arriving at the youngest non-inoculated true leaves first and the oldest leaves last (Holmes, 1930; Maule and Palukaitis, 1991; Hull, 2014; DeMell et al., 2023). In our study, in the inoculated true leaves of older systemically virus-infected and virus-mock-inoculated plants of the three cultivars, the severity of fungal disease symptoms was greatest on the oldest leaf (leaf 2) but then declined with decreasing leaf age, culminating in complete symptom absence on the youngest inoculated leaf (leaf 7). In younger plants of the fully fungus-susceptible cultivar and the cultivar with SDGR, this decline was evident between the oldest leaf (leaf 2) and next oldest leaf (leaf 3), while symptoms rarely developed on the next youngest leaf (leaf 4) and never on the youngest inoculated leaf (leaf 5). Therefore, this decline in fungal leaf severity was closely associated with leaf maturity. Moreover, in the older plants of all three cultivars and the younger plants of the cultivars lacking host fungal resistance or carrying SDGR, this decline was also closely related to increasing VC reflecting systemic movement from inoculated cotyledons via the phloem to the youngest non-inoculated true leaves first and the oldest non-inoculated true leaves last.

The inverse relationship between fungal disease severity and VC across leaf positions reveals a complex interplay between host development, viral pathology, and genetic pathogen resistance. In youngest leaves, high metabolic sink activity drove rapid virus multiplication. When this intense viral load was present, fungal disease remained at zero or minimal levels in plants of different ages (i.e., 1.05 and 1.07 SBGS, respectively). This suggested that within young tissues viral multiplication triggered a transient, non-specific systemic immune priming that restricted initial fungal colonization. However, as leaves matured and VC diminished, the host plant’s genetic defense system became active. In the cultivar with SDGR, this effectively suppressed non-RB fungal strain disease compared with its expression in the fully fungus-susceptible cultivar. In contrast, where host resistance relied on PGR and TSSIR, although a similar VC decline occurred, fungal disease was more tightly constrained, especial after the 0–3 dai second fungal inoculation. This suggested that fungal disease expression was not a simple developmental source–sink effect. It instead represented a developmentally gated signaling interaction where virus infection likely interfered with or modulated the SA/JA cross-talk required for optimal SDGR and PGR expression, with the intensity of this interference dictated by leaf position, plant age, and viral load. However, a combination of virus-induced nutrient leakage and tissue damage can also assist invading fungal pathogen entry and enhance its nutrition (Handford and Carr, 2007; Hull, 2014; Tungadi et al., 2017). Nevertheless, where system overload occurs, this can prioritize resources for an already established virus over an invading fungus (Huot et al., 2014). In addition, a physical barrier that involves the formation of callose structures around fungal infection foci can develop (Liu et al., 2018; German et al., 2023; Wang et al., 2021), yet viral-mediated induction of host callose-degrading enzymes can break down these cell-wall defenses, compromising the physical barrier to facilitate subsequent fungal entry and colonization (Beffa et al., 1996; Liu et al., 2021).

Four major highlights relevant to host resistance/susceptibility arose from the study of Abidin et al. (2025). These resembled the key outcomes of this study as listed in **Table 3**, but there were also differences. Their key findings were as follows: (a) “Strong fungal disease suppression occurred when a mixture of RB and non-RB fungal strains infected cotyledons of a canola cultivar with fungal SDGR but only weak suppression when the cultivar had fungal PGR”. In our study, when the true leaves of cultivars with virus-mock-inoculated cotyledons were inoculated with a mixture of RB and non–RB fungal strains, there were both similarities and differences. In the cultivar with SDGR, the same result occurred in leaf 2 in both younger and older plants when the same fungal strain mixtures and the RB strain alone were present, but the non-RB alone strain failed to cause any infection. In the cultivar with PGR, in leaf 2 of older plants infection still occurred but at low levels regardless of which fungal strain or strain combination was inoculated, whereas in younger plants no infection whatsoever occurred regardless of whether any of these fungal strains were inoculated in combination or alone. Thus, in the younger plants, SDGR was more effective against the non-RB strain, whereas PGR was effective regardless of fungal strain or strain combination inoculated. (b) “Fungal disease suppression by systemic virus infection when fungal resistance was lacking or PGR and TSSIR were present, and virus was inoculated to cotyledons before either fungal strain”. Here what transpired resembled what occurred in the cultivar lacking fungal resistance where fungal disease severity was suppressed in coinfected true leaves (especially leaf 2) compared with leaves infected with the fungus alone regardless of the strain combination sequence, but not in the cultivar with PGR and TSSIR where the low fungal disease severity values in older plants, although greater at 0–0 than at 0–3 dai, were always similar regardless of fungal strain combination and the presence or absence of the virus. (c) “Suppression of VC in young systemically infected leaves of cultivars with SDGR or lacking any fungal resistance when the fungal inoculation to entire cotyledons was with either of its strains or to cotyledon halves with the non-RB strain, but not with the RB strain in the cultivar lacking fungal resistance”. Here their entire cotyledon inoculation situation more closely resembled the VC scenario that developed in true leaves of systemically virus-infected plants of the fully fungus-susceptible cultivar where the two fungal strain combinations with its non-RB strain inoculated first suppressed VC compared with when the virus was present alone, whereas, instead, their half cotyledon inoculation situation most resembled the VC scenario where the two fungal strain combinations with its RB strain inoculated first stimulated VC to reach higher levels than when the virus was present alone. By contrast, in the systemically infected plants of the cultivar with SDGR, their entire cotyledon situation more closely resembled the VC scenario that developed where VC was suppressed to the same extent by the three strain combinations that included the RB strain, but not by the strain combination that only included the non-RB strain which developed the same VC as when the virus was present alone. (d) “Increase of VC values in young systemically virus-infected leaves of the cultivar with TSSIR and fungal PGR when the second cotyledon inoculation was with either fungal strain”. Here there was considerable suppression of VC in this cultivar in comparison with the much higher VC in the fully fungus-susceptible cultivar infected with the virus alone. Uniformly low VC values developed in its systemically infected leaves regardless of whether they came from plants with true leaves coinfected with different fungal strain combinations or infected with the virus alone.

In this study, when virus-mock-inoculated or virus-infected plants of the fully fungus-susceptible cultivar were inoculated with the fungus, virus presence suppressed fungal disease regardless of its strain combination, with this suppression being greater in younger than older plants. In addition, when virus-mock-inoculated or virus-infected plants of the cultivar with SDGR were inoculated with the fungus, virus presence suppressed the fungal disease caused by its RB strain, with this effect showing in the younger plants. Reciprocal outcomes in terms of fungal disease severity and VC resulted from inoculation of different combinations of RB or non-RB fungal strains to true leaves of different maturity growing on systemically virus-infected or cotyledon virus-mock-inoculated plants of cultivars with different pathogen resistances or susceptibilities. In the cultivar with SDGR, its presence decreased fungal disease severity on the lower true leaves of systemically virus-infected or cotyledon virus-mock-inoculated plants infected with the fungal non-RB strain regardless of whether it was present alone or in two different combinations with the fungal non-RB strain. Conversely, the presence of the RB fungus strain decreased VC in true leaves of coinfected plants regardless of whether it was present alone or in the same two combinations with the fungal RB strain. Since the complete suppression of the non-RB strain [RB (first)–RB (second) combination] by SDGR gene *Lep3* (Li and Cowling, 2003) in the older plants (i.e., at 1.07 SBGS) was overcome when the virus was present, this suggests an antagonistic interaction where virus infection interferes with resistance gene effectiveness. Abidin et al. (2025) found the same thing regarding the expression of fungal disease severity when, in the absence of the virus, the same combinations of these two fungal strains were inoculated in these same sequences to cotyledons of the cultivar with SDGR. Similar interactions between these two fungal strains in the presence of SDGR but the absence of the virus were reported earlier (Li et al., 2004, Li et al., 2005, Li et al., 2006a). A possible explanation for the decrease in VC values when the RB fungal strain was present in the cultivar with SDGR is that this strain may have activated SAR which diminished VC not only within coinfected lower leaves but also remotely within younger upper leaves in which the fungus was absent. With the fully fungus-susceptible cultivar, the presence of the non-RB fungal strain decreased VC in the leaves of coinfected plants when the non-RB strain was inoculated first but increased it when the RB strain was inoculated first. The entire cotyledon inoculation situation of Abidin et al. (2025) resembled the VC scenario that developed in plants of the fully fungus-susceptible cultivar when the fungal non-RB strain was inoculated first (VC suppressed), whereas their half cotyledon inoculation situation resembled the VC scenario that occurred in plants of this cultivar when the fungal RB strain was inoculated first (VC increased). In this cultivar, the unrestricted hyphal invasion capacity of the fungal RB strain enabling it to spread from leaf-to-stem (Li et al., 2006a) may have blocked SAR from entering the vascular system and moving to the upper leaves, thereby preventing VC suppression. Such an increase in VC was reported previously where *Phaseolus vulgaris* (French bean) leaves were inoculated first with *Fusarium oxysporum* f. sp. *phaseoli* and 24 h later with sunn-hemp mosaic virus (genus *Tobamovirus*, family *Virgaviridae*) (Gbaja and Chant, 1985). The resulting coinfection almost doubled VC in systemically virus-infected plants compared to single-pathogen infection with this virus. In that era, it was suggested that the presence of this fungus actively changes metabolic reactions in the plant tissue, making it more favorable for virus multiplication (Chant and Bateman, 1981; Gbaja and Chant, 1985).

In the cultivar with combined fungal PGR and viral TSSIR, when infected solely by the fungal RB strain, fungal disease was entirely absent in younger plants or achieved much lower severity than in the fully fungus-susceptible cultivar or the cultivar with SDGR in older plants. Moreover, in contrast to what occurred in older plants of the other two cultivars, its fungal disease values in virus-infected leaves 2 and 3 were similar to those in these two leaves on plants lacking any virus infection. Furthermore, with this cultivar, there were no significant differences between the low VC values resulting from systemic virus infection without fungal infection in younger plants or when fungal infection was also present in older plants. The suppressed fungal disease values that developed in older plants can be attributed almost entirely to PGR because they were similar to each other regardless of the presence or absence of the virus. However, in younger plants, both PGR and TSSIR might have been responsible for the fungus being absent, or its younger growth might have been more resistant to fungal infection, as what occurred in leaves 5 and 7 which never developed any fungal disease symptoms in any of the three cultivars regardless of plant age. The suppressed VC values that developed in this cultivar can be attributed entirely to TSSIR in younger plants as the fungus was absent and almost entirely to TSSIR in older plants because they were similar regardless of the presence or absence of the fungus. When the higher fungal disease severities that developed on plants of the fungus-susceptible cultivar or on the cultivar with SDGR when the fungal RB strain was present alone were compared with much lower values on the cultivar with PGR and TSSIR, these values were higher in older than younger plants, although still at a much lower level. This is not surprising as factors such as plant maturity, leaf tissue maturation, and plant–pathogen interactions can change fungal resistance reactions (e.g., Develey-Rivière and Galiana, 2007). Establishment, disruption, and breakdown of fungal resistance can occur at different plant ages and growth stages, such as at transition to adult plant and flowering transition as well as at the onset of senescence (Develey-Rivière and Galiana, 2007; Hu and Yang, 2019; DeMell et al., 2023)—for example, in relation specifically to fungal resistance, Li et al. (2006b) demonstrated how, at a higher temperature regime of 18/24°C, the expression of resistance between seedlings and adult plants could not be distinguished at inoculation for nearly all growth stages. In contrast, at a lower temperature regime of 11/18°C, this relationship was absent for inoculations to younger plants. Moreover, another study by Li et al. (2006a) investigated the interactions arising from co-inoculation with the same fungal non-RB and RB strains where, on its own, the former strain induced localized necrotic lesions (resistant phenotype) on cotyledons of the cultivar with SDGR while the later induced large spreading necrotic lesions (fully fungus susceptible phenotype), yet when applied in mixtures to cotyledons of the same cultivar only localized necrotic lesions developed.

Concerning differences due to second fungal inoculation times at 0–0 than at 0–3 dai, for the fully fungus-susceptible cultivar and the cultivar with PGR and TSSIR, with very few exceptions, the lower true leaves (this study) or cotyledons (Abidin et al., 2025) that were lacking virus infection but inoculated with different fungal strain combinations developed higher fungal disease severity values when they were inoculated at 0–0 than at 0–3 dai. In the study of Abidin et al. (2025), the exceptions were cases where there was a minimal change in fungal disease severity between inoculations at 0–0 and 0–3 dai for the fungal strain combination non-RB (first):RB (second). In the current study, this exception occurred only in older plants of the cultivar lacking fungal resistance both where the fungus was present alone and where there was coinfection. For the cultivar with SDGR, when the lower leaves on older plants that were coinfected or without virus infection were inoculated with different fungal strain combinations they always developed higher fungal disease values at 0–0 dai than at 0–3 dai, but only clearly did so in five of 10 instances in younger plants. For Abidin et al. (2025) in this cultivar, where solely the fungus was present, with the fungal strain combination RB (first):RB (second), there was a sharp decline in fungal disease between inoculations at 0–0 and 0–3 dai, but only minor declines otherwise. Kuć (1987) suggested that infecting a plant with a non-RB strain elicits a fast response that increases a plant’s defenses preventing wider colonization afterwards (Li et al., 2006a). Abidin et al. (2025) suggested that diminished fungal disease severity on cotyledons was due to the activation of a SAR-like defensive response against the fungus when its non-RB strain was inoculated first and the RB strain was inoculated at 0–3 dai (Abidin et al., 2025). A further possible explanation could be that the RB strain might have circumvented the resistance reaction that restricts hyphal growth and fungal reproduction following a prior fungal inoculation (Staskawicz et al., 1995; Dangl et al., 1996).

For VC, in the current study, the only times when there was a change between second fungal inoculation times was in the cultivar lacking fungal resistance where it increased between second fungal inoculations at 0–0 and 0–3 dai for the two fungal strain combinations in which the non-RB strain was placed first in younger plants and for the two strain combinations in which the RB strain was placed first in older plants. In the study of Abidin et al. (2025), following the initial virus inoculation of the cultivar lacking fungal resistance and the cultivar with PGR + TSSIR, VC only increased by a very small amount between fungal or viral inoculations at 0–0 and 0–3 dai. Conversely, in the cultivar with SDGR, with one exception in which there was no change (non-RB fungal strain inoculated alone), it declined instead. Abidin et al. (2025) explained the decline in VC between second virus inoculations at 0–0 and 0–3 dai in terms of the “superinfection exclusion” phenomenon whereby an initial infection by one viral strain prevents or interferes with a subsequent infection by a similar strain of the same virus (Nunna et al., 2023).

Using “superinfection exclusion” in a cross-kingdom (fungus–virus) context overlooks critical physical and metabolic dynamics unique to fungal pathogenesis. Beyond providing a localized spatial blockade, fungal pathogen presence can restrict viral accumulation by aggressive host resource sequestration. Fungal tissues act as metabolic sinks that redirect the host’s allocation of photo-assimilates away from normal cellular processing (Berger et al., 2007). Because plant viruses rely entirely on the host cell’s active translation machinery, energetic currency (adenosine triphosphate) and free nucleotide/amino acid pools for replication (Hipper et al., 2013), this severe nutrient “sinking” by fungal structures starves the local environment, reducing the cellular capacity required to sustain viral replication at a high rate. Concurrently, the spatial constraints imposed by fungal colonization can impede systemic viral translocation. To establish a systemic infection, viral particles must successfully load into, navigate through, and unload from the host’s vascular transport system (Hipper et al., 2013). However, hyphal proliferation and the subsequent development of localized fungal lesions cause significant physical damage to the vascular tissue architecture (Malinowski et al., 2024). Moreover, viral long-distance movement is contingent upon uninterrupted cellular networks and functional transit structures (Hipper et al., 2013). Therefore, the host’s own defensive structural responses to fungal invasion (e.g., callose deposition) occlude these conductive channels (Riseh et al., 2025). This disruption of fluid dynamics and physical narrowing creates a powerful structural barrier that restricts long-distance virus movement through the infected plant. However, this phenomenon cannot explain the VC findings where there was no second virus inoculation in both the study of Abidin et al. (2025) and this study. There was no difference between what occurred with second fungal inoculations at 0–0 and 0–3 dai, and VC increased rather than declined. Furthermore, there was no reciprocal pattern between fungal disease severity and VC in any of the three cultivars. Further research is needed to understand the reasons for the alterations in fungal disease severity and VC that occur following second fungal inoculations at 0–0 and 0–3 dai.

Our findings emphasize the importance of taking into account the common occurrence of more than one pathogen infecting the same plant within commercial crops when deploying cultivars with single-pathogen resistances as their effectiveness may be altered. They also underscore the necessity of knowing the most appropriate leaf age and position and plant growth stage to choose when assessing cultivars, breeding lines, or germplasm accessions for pathogen resistances that may assist with breeding new cultivars with more effective resistance to pathogen mixtures. More widely, our findings offer an important foundational step toward developing more integrated and effective disease management approaches that include combinations of the best available host resistance, phytosanitary and cultural control measures for situations where pathogen coinfection causes diseases that amplify crop losses. In conclusion, our research contributes toward improving evidence-based approaches to breeding plant cultivars with combined viral and fungal pathogen resistances and devising integrated plant disease management strategies that operate effectively against virus–fungus plant pathogen mixtures. This will help toward the provision of more sustainable and effective crop protection strategies in the face of evolving pathogen coinfection pressures, including encouraging plant breeding programs to pyramid multiple pathogen resistance genes that remain robust against complex coinfections.

## Data Availability

The raw data supporting the conclusions of this article will be made available by the first author, without undue reservation.
