# Supplementary material for: Alterations to fungal and viral pathogen infection in coinfected plant cultivars with diverse single-pathogen host resistances
Source: Front Plant Sci. 2026 Sep 9;17:1809771. doi: 10.3389/fpls.2026.1809771 (PMC13597726; doi:10.3389/fpls.2026.1809771)
Supplement: Supplementary file 1 [file Table1.docx]

| **Source of variation** | **d.f.** | **v.r.** | **F pr** | **LSD_(0.05)_** |
| --- | --- | --- | --- | --- |
| Cultivar | 1 | 561 | <.001 | 1.34 |
| +/- virus | 1 | 224 | <.001 | 1.34 |
| Fungus isolate sequence | 3 | 38 | <.001 | 1.89 |
| Leaf position | 2 | 372 | <.001 | 1.64 |
| Inoculation time | 1 | 179 | <.001 | 1.34 |
| Cultivar x +/- virus | 1 | 51 | <.001 | 1.89 |
| Cultivar x fungus isolate sequence | 3 | 34 | <.001 | 2.68 |
| +/- virus x fungus isolate sequence | 3 | 3 | <.01 | 2.68 |
| Cultivar x leaf position | 2 | 92 | <.001 | 2.32 |
| +/- virus x leaf position | 2 | 19 | <.001 | 2.32 |
| Fungus isolate sequence x leaf position | 6 | 6 | <.001 | 3.28 |
| Cultivar x inoculation time | 1 | 52 | <.001 | 1.89 |
| +/- virus x inoculation time | 1 | 9 | <.01 | 1.89 |
| Fungus isolate sequence x inoculation time | 3 | 19 | <.001 | 2.68 |
| leaf position x inoculation time | 2 | 20 | <.001 | 2.32 |
| Cultivar x +/- virus x fungus isolate sequence | 3 | 16 | <.001 | 3.79 |
| Cultivar x +/- virus x leaf position | 2 | 9 | <.001 | 3.28 |
| Cultivar x fungus isolate sequence x leaf position | 6 | 4 | <.001 | 4.64 |
| +/- virus x fungus isolate sequence x leaf position | 6 | 1 | 0.53 | 4.64 |
| Cultivar x +/- virus x inoculation time | 1 | 13 | <.001 | 2.68 |
| Cultivar x fungus isolate sequence x inoculation time | 3 | 3 | <.05 | 3.79 |
| +/- virus x fungus isolate sequence x inoculation time | 3 | 5 | <.01 | 3.79 |
| Cultivar x leaf position x inoculation time | 2 | 9 | <.001 | 3.28 |
| +/- virus x leaf position x inoculation time | 2 | 2 | 0.11 | 3.28 |
| Fungus isolate sequence x leaf position x inoculation time | 6 | 2 | 0.14 | 4.64 |
| Cultivar x +/- virus x blackleg isolate interaction x leaf position | 6 | 2 | 0.15 | 6.56 |
| Cultivar x +/- virus x blackleg isolate interaction x inoculation time | 3 | 5 | 0.10 | 5.36 |
| Cultivar x +/- virus x leaf position x inoculation time | 2 | 1 | 0.45 | 4.64 |
| Cultivar x blackleg isolate interaction x leaf position x inoculation time | 6 | <1 | 0.97 | 6.56 |
| +/- virus x blackleg isolate interaction x leaf position x inoculation time | 6 | 4 | <.01 | 6.56 |
| Cultivar x +/- virus x fungus isolate sequence x leaf position x inoculation time | 6 | <1 | 0.44 | 9.28 |

**Table 1a: Statistical outcomes in relation to the *Leptosphaeria maculans* % leaf disease indices at the 1.05 Sylvester-Bradley growth stage**

**Table 1b: Statistical outcomes in relation to the *Leptosphaeria maculans* % leaf disease indices at the 1.07 Sylvester-Bradley growth stage**

| **Source of variation** | **d.f.** | **v.r.** | **F pr** | **LSD_(0.05)_** |
| --- | --- | --- | --- | --- |
| Cultivar | 2 | 1062 | <.001 | 0.8 |
| +/- virus | 1 | 89 | <.001 | 0.6 |
| Fungus isolate sequence | 3 | 92 | <.001 | 0.9 |
| Leaf position | 4 | 994 | <.001 | 1.0 |
| Inoculation time | 1 | 330 | <.001 | 0.6 |
| Cultivar x +/- virus | 2 | 180 | <.001 | 1.1 |
| Cultivar x fungus isolate sequence | 6 | 39 | <.001 | 1.5 |
| +/- virus x fungus isolate sequence | 3 | 8 | <.001 | 1.2 |
| Cultivar x leaf position | 8 | 243 | <.001 | 1.7 |
| +/- virus x leaf position | 4 | 18 | <.001 | 1.4 |
| Fungus isolate sequence x leaf position | 12 | 10 | <.001 | 2.0 |
| Cultivar x inoculation time | 2 | 14 | <.001 | 1.1 |
| +/- virus x inoculation time | 1 | <1 | 0.55 | 0.9 |
| Fungus isolate sequence x inoculation time | 3 | 2 | 0.07 | 1.2 |
| leaf position x inoculation time | 4 | 27 | <.001 | 1.4 |
| Cultivar x +/- virus x blackleg isolate interaction | 6 | 7 | <.001 | 2.2 |
| Cultivar x +/- virus x leaf position | 8 | 24 | <.001 | 2.4 |
| Cultivar x blackleg isolate interaction x leaf position | 24 | 6 | <.001 | 3.4 |
| +/- virus x blackleg isolate interaction x leaf position | 12 | 1 | 0.91 | 2.8 |
| Cultivar x +/- virus x inoculation time | 2 | 30 | <.001 | 1.5 |
| Cultivar x blackleg isolate interaction x inoculation time | 6 | 3 | <.01 | 2.2 |
| +/- virus x blackleg isolate interaction x inoculation time | 3 | 1 | 0.60 | 1.8 |
| Cultivar x leaf position x inoculation time | 8 | 5 | <.001 | 2.4 |
| +/- virus x leaf position x inoculation time | 4 | 3 | <.05 | 2.0 |
| Blackleg isolate interaction x leaf position x inoculation time | 12 | <1 | 1.00 | 2.8 |
| Cultivar x +/- virus x blackleg isolate interaction x leaf position | 24 | 1 | 0.77 | 4.8 |
| Cultivar x +/- virus x blackleg isolate interaction x inoculation time | 6 | <1 | 0.90 | 3.1 |
| Cultivar x +/- virus x leaf position x inoculation time | 8 | 4 | <.001 | 3.4 |
| Cultivar x fungus isolate sequence x leaf position x inoculation time | 24 | <1 | 1.00 | 4.8 |
| +/- virus x fungus isolate sequence x leaf position x inoculation time | 12 | <1 | 1.00 | 3.9 |
| Cultivar x +/- virus x fungus isolate sequence x leaf position x inoculation time | 24 | <1 | 1.00 | 6.8 |

| **Source of variation** | **d.f.** | **v.r.** | **F pr** | **LSD_(0.05)_** |
| --- | --- | --- | --- | --- |
| Cultivar | 2 | 9701 | <.001 | 0.03 |
| Fungus isolate sequence | 4 | 2226 | <.001 | 0.04 |
| Leaf position | 3 | 51913 | <.001 | 0.03 |
| Fungal inoculation time | 1 | 1079 | <.001 | 0.02 |
| Cultivar x fungus isolate sequence | 8 | 2830 | <.001 | 0.06 |
| Cultivar x leaf position | 6 | 3440 | <.001 | 0.06 |
| Fungus isolate sequence x leaf position | 12 | 140 | <.001 | 0.07 |
| Cultivar x fungal inoculation time | 2 | 416 | <.001 | 0.04 |
| Fungus isolate sequence x fungal inoculation time | 4 | 4 | <.01 | 0.05 |
| leaf position x fungal inoculation time | 3 | 335 | <.001 | 0.05 |
| Cultivar x fungus isolate sequence x leaf position | 24 | 152 | <.001 | 0.13 |
| Cultivar x fungus isolate sequence x fungal inoculation time | 8 | 3 | <.001 | 0.09 |
| Cultivar x leaf position x fungal inoculation time | 6 | 191 | <.001 | 0.08 |
| Fungus isolate sequence x leaf position x fungal inoculation time | 12 | 12 | <.001 | 0.10 |
| Cultivar x fungus isolate sequence x leaf position x fungal inoculation time | 24 | 11 | <.001 | 0.18 |

**Table 2a: Statistical outcomes in relation to the virus concentration [Exponentially transformed optical density A_405_ values (ELISA)] at the 1.05 Sylvester-Bradley growth stage**

**Table 2b: Statistical outcomes in relation to the virus concentration [Exponentially transformed optical density A_405_ values (ELISA)] at the 1.07 Sylvester-Bradley growth stage**

| **Source of variation** | **d.f.** | **v.r.** | **F pr** | **LSD_(0.05)_** |
| --- | --- | --- | --- | --- |
| Cultivar | 2 | 10797 | <.001 | 0.05 |
| Fungus isolate sequence | 4 | 2926 | <.001 | 0.06 |
| Leaf position | 5 | 68299 | <.001 | 0.07 |
| Fungal inoculation time | 1 | 320 | <.001 | 0.04 |
| Cultivar x fungus isolate sequence | 8 | 3780 | <.001 | 0.11 |
| Cultivar x leaf position | 10 | 2301 | <.001 | 0.12 |
| Fungus isolate sequence x leaf position | 20 | 249 | <.001 | 0.15 |
| Cultivar x fungal inoculation time | 2 | 648 | <.001 | 0.07 |
| Fungus isolate sequence x fungal inoculation time | 4 | 31 | <.001 | 0.09 |
| leaf position x fungal inoculation time | 5 | 358 | <.001 | 0.10 |
| Cultivar x fungus isolate sequence x leaf position | 40 | 318 | <.001 | 0.27 |
| Cultivar x fungus isolate sequence x fungal inoculation time | 8 | 93 | <.001 | 0.15 |
| Cultivar x leaf position x fungal inoculation time | 10 | 426 | <.001 | 0.17 |
| Fungus isolate sequence x leaf position x fungal inoculation time | 20 | 41 | <.001 | 0.22 |
| Cultivar x Fungus isolate sequence x leaf position x fungal inoculation time | 40 | 35 | <.001 | 0.38 |
